# TRAF4 Is a Novel Phosphoinositide-Binding Protein Modulating Tight Junctions and Favoring Cell Migration

**DOI:** 10.1371/journal.pbio.1001726

**Published:** 2013-12-03

**Authors:** Adrien Rousseau, Alastair G. McEwen, Pierre Poussin-Courmontagne, Didier Rognan, Yves Nominé, Marie-Christine Rio, Catherine Tomasetto, Fabien Alpy

**Affiliations:** 1 Institut de Génétique et de Biologie Moléculaire et Cellulaire (IGBMC), Functional Genomics and Cancer Department, Illkirch, France; 2 Institut National de la Santé et de la Recherche Médicale (INSERM), U 964, Illkirch, France; 3 Centre National de la Recherche Scientifique (CNRS), UMR 7104, Illkirch, France; 4 Université de Strasbourg, Illkirch, France; 5 IGBMC, Structural Biology and Genomics Platform, Illkirch, France; 6 Laboratory of Therapeutic Innovation, CNRS UMR 7200, Medalis Drug Discovery Center, Illkirch, France; 7 Group Oncoproteins, Institut de Recherche de l'Ecole de Biotechnologie de Strasbourg, CNRS UMR 7242, BP 10413, Illkirch, France; Max Planck Institute of Molecular Cell Biology and Genetics, Germany

## Abstract

Tumor necrosis factor (TNF) receptor-associated factor 4 (TRAF4) is frequently overexpressed in carcinomas, suggesting a specific role in cancer. Although TRAF4 protein is predominantly found at tight junctions (TJs) in normal mammary epithelial cells (MECs), it accumulates in the cytoplasm of malignant MECs. How TRAF4 is recruited and functions at TJs is unclear. Here we show that TRAF4 possesses a novel phosphoinositide (PIP)-binding domain crucial for its recruitment to TJs. Of interest, this property is shared by the other members of the TRAF protein family. Indeed, the TRAF domain of all TRAF proteins (TRAF1 to TRAF6) is a *bona fide* PIP-binding domain. Molecular and structural analyses revealed that the TRAF domain of TRAF4 exists as a trimer that binds up to three lipids using basic residues exposed at its surface. Cellular studies indicated that TRAF4 acts as a negative regulator of TJ and increases cell migration. These functions are dependent from its ability to interact with PIPs. Our results suggest that TRAF4 overexpression might contribute to breast cancer progression by destabilizing TJs and favoring cell migration.

## Abstract 2

The cancer-associated TRAF4 protein has a TRAF domain that is a bona fide phosphoinositide-binding domain and involved in the modulation of tight junctions and cell migration.

## Author Summary

Tumor necrosis factor (TNF) receptor-associated factor 4, also known as TRAF4, is an unusual member of the TRAF protein family. While TRAFs are primarily known as regulators of inflammation, antiviral responses, and apoptosis, research on TRAF4 has identified its involvement in development and cancer. Importantly TRAF4 overexpression has been associated with a poor prognosis in breast cancers. TRAF4 has multiple subcellular localizations: to the plasma membrane in tight junctions (TJs), cytoplasmic and nuclear. The recruitment mechanisms and the functional impact of these distinct localizations are not completely understood. Here we investigate how TRAF4 is recruited to TJs and its involvement in cell–cell contacts in mammary epithelial cells (MECs). We show that the TRAF domain of all TRAFs contains a lipid binding module, and that TRAF4 uses this domain to form a trimeric complex that associates with phosphoinositides at the plasma membrane. Moreover this interaction is necessary for its recruitment to TJs. Additionally, we show that through its interaction with lipids TRAF4 delays TJ assembly and increases cell migration. We propose that TRAF4 has an important function in cancer progression by destabilizing TJs and favoring cell migration.

## Introduction

*Tumor necrosis factor receptor-associated factor 4* (*TRAF4*) was originally identified as a gene overexpressed in breast carcinoma [1],[2]. Interestingly, *TRAF4* overexpression is not restricted to breast cancer and extends to a variety of different carcinomas [3],[4]. TRAF4 belongs to the TRAF family that is composed of seven members in humans [5],[6]. Among the seven TRAF family members, TRAF4 is one of the most conserved during evolution [7]. Indeed, a TRAF4 ortholog has already been identified in snail fur (Hydractinia achinata), a cnidaria [8]. Moreover, the unique TRAF protein in the worm shares a higher homology with human TRAF4 than with other human TRAF proteins [9]. Furthermore, one of the three fly TRAF proteins, dTRAF1, shares the highest homology with human TRAF4 [9],[10]. In line with an essential and conserved biological function of TRAF4, flies that carry null-alleles of TRAF4 have many developmental abnormalities, leading to lethality before the pupal stage [11],[12]. Likewise, TRAF4 deficiency in mice was lethal at the embryonic stage in approximately one third of the homozygote mutants [13]. All surviving animals exhibited multiple defects including trachea alteration and various nonfully penetrant phenotypes involving the axial skeleton and the central nervous system [13],[14]. Surviving adult TRAF4-deficient mice also exhibited ataxia, associated with myelination alteration [15]. Together, these various genetic models suggest that TRAF4 has an essential function conserved in most, if not all, pluricellular animals.

TRAF4 encodes a 53 kDa adaptor protein with multiple subcellular localizations. Indeed, cytoplasmic, nuclear, and membrane localizations have been described in the literature [1],[16],[17]. Of interest, the subcellular localization of TRAF4 is altered in cancers. While in normal breast tissue the protein is predominantly localized in the plasma membrane [18], more precisely in tight junctions (TJs) present at the apical membrane of polarized epithelial cells [19], in cancer samples the protein can be localized either in the cytoplasm and/or in the nucleus of cancer epithelial cells [1],[3],[16]. Until recently the implication of these multiple localizations were unclear. A recent report shed light into the significance of the nuclear localization of TRAF4 in breast cancers [20]. This study indicated that TRAF4 nuclear localization in breast tumors was associated with poor survival in breast cancer patients after adjuvant therapy. Moreover, this report showed that TRAF4 promotes p53 protein destabilization in the nucleus of cancer cells and contributes to resistance to cytotoxic stress in cancer cells [20]. To date, the function of TRAF4 in TJs and in the cytoplasm remains unclear. Many lines of evidence indicate that TRAF4 functions in the organization and patterning of the cell cortex [12],[21]. TRAF4 is required for the polarized trafficking of NADPH oxidase to filopodia in migrating endothelial cells [22],[23]. In the fly, TRAF4 can interact with proteins involved in asymmetric division [21] and functions in the establishment of the junctional architecture of mesodermal cells [12]. In the mouse nervous system, TRAF4 deficiency altered the formation and/or stability of axoglial and interglial junctions [15]. Collectively, all these data indicate that TRAF4 might exert a function related to cell junctions and polarity [24]. However, TRAF4 appears to function in a cell-specific manner, making its functional characterization difficult. While TRAF4 is ubiquitously expressed at a basal level, there exists a spatial and temporal up-regulation of TRAF4 gene expression during specific developmental stages [25],[26]. For example, during gastrulation in frogs, mRNA encoding TRAF4 and one of its interacting partners SMURF1 become enriched in the neural plate and neural crest cells. In these cells, both TRAF4 and SMURF1 are essential for proper neural crest development and neural plate morphogenesis [25]. In human mammary epithelial cells (MECs), the role of TRAF4 in TJs remains unclear. However, we showed previously that this localization was a highly dynamic process, supporting the notion that TRAF4 might relay signals from the cell membrane to the cytoplasm, and possibly the nucleus [17]. Given the recently established role of nuclear TRAF4 in the destabilization of the tumor suppressor p53 protein [20], it is now necessary to understand how TRAF4 subcellular localization in the mammary epithelium is regulated under physiopathological conditions. To address this, we explored the molecular determinants involved in its subcellular localization at the plasma membrane and in TJs of MECs. We also directly addressed the function of TRAF4 in the formation and maintenance of TJs in polarized cells. Finally, having established that TRAF4 is a negative regulator of TJs in breast cells, we have explored its contribution to cell motility.

## Results

### TRAF4 Is a Negative Regulator of TJs

**Figure 1:**
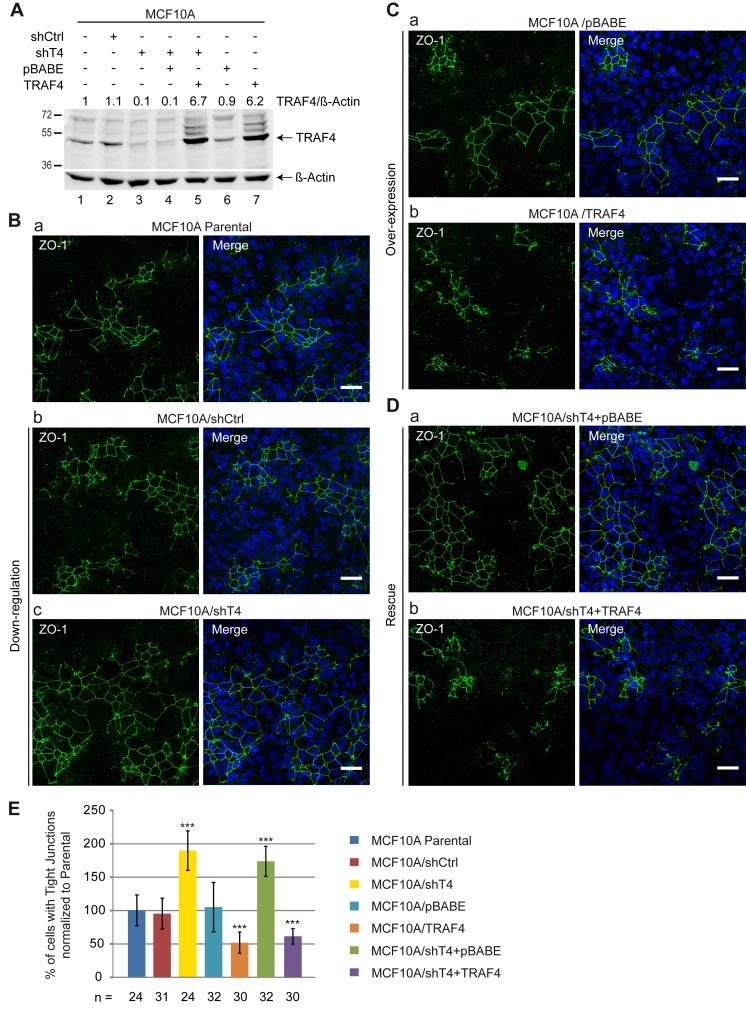
TRAF4 protein level modulates TJs in confluent MCF10A monolayers. (A) Western-blot analysis of TRAF4 in parental and in established MCF10A cell lines. To knock down TRAF4, parental MCF10A cells (lane 1) were transduced with a shRNA targeting TRAF4 (MCF10A/shT4, lanes 3–5); a nonspecific shRNA was used as control (MCF10A/shCtrl, lane 2). To restore TRAF4 expression, MCF10A/shT4 cells were transduced with a shT4-insensitive vector encoding TRAF4 (MCF10A/shT4+TRAF4, lane 5); the control cell line (MCF10A/shT4+pBABE, lane 4) was transduced with the empty vector. A gain of function and a control cell line were generated with a TRAF4 expression plasmid (MCF10A/TRAF4, lane 7) or the empty vector (MCF10A/pBABE, lane 6), respectively. TRAF4 expression levels normalized to actin are indicated. (B–D) The presence of TJs was estimated by ZO-1 staining in the different cell lines of TRAF4 loss of function (B), gain of function (C), and rescue (D) experiments. Left panels are representative confocal sections of ZO-1 staining (green), and right panels are merges with Hoechst staining (blue). Scale bar, 20 µm. (E) TJ quantification. Score representing the number of cells with a continuous ZO-1 staining, normalized to parental MCF10A cells (percentage). The number of microscopic fields used for the quantification is indicated at the bottom of the bar chart. TRAF4 knock-down increased the number of cells with TJs, whereas TRAF4 overexpression had the opposite effect.

MECs form ducts and acini with an established apicobasal polarity [27]. Adhesion and TJ formation are instrumental to the establishment of this polarity [28],[29]. In polarized MECs, TRAF4 is mainly found in TJs [17], suggesting a specific function of the protein in the formation and/or dissociation of TJs. To address the function of TRAF4 in TJs, we used the human immortalized normal MEC model, MCF10A [30]. Monolayers of MCF10A provide a good system to study TJs because at confluence they form clusters of polarized cells exhibiting TJs and they can be used to measure the contribution of a given gene in the formation and/or stabilization of TJs [31],[32]. In practice, quantification of cell clusters exhibiting a continuous membrane-associated ZO-1 staining indicates the extent of cells making TJs. This cell system has been successfully used to demonstrate TJ alterations following long-term TGF-β treatment [31]. To study the role of TRAF4 on TJs, we down-regulated its expression in MCF10A cells using a shRNA strategy (Figure 1A). In cells stably expressing a shRNA specific for TRAF4 (MCF10A/shT4), TRAF4 expression was reduced to less than 10% compared to parental MCF10A cells and to cells expressing a control shRNA (MCF10A/shCtrl). Next, MCF10A/shT4, MCF10A/shCtrl, and MCF10A parental cells were plated at the same density, and 48 h later, cell monolayers were fixed and stained for the TJ marker ZO-1 (Figure 1B). The relative number of cells harboring TJs was then measured. Cells harboring TJs were defined as cells with an enclosed ring of contiguous apical ZO-1 staining. Compared to parental MCF10A and to MCF10A/shCtrl cell lines, TRAF4 silencing was associated with a 2-fold increase in cells harboring TJs (Figure 1B,E).

To support the role of TRAF4 expression on TJs, we performed the complementary experiment in which TRAF4 was overexpressed in MCF10A cells. In cells stably expressing the protein (MCF10A/TRAF4), TRAF4 was increased over 6-fold as compared to parental MCF10A cells and to cells transduced with the empty vector (MCF10A/pBABE). The presence of TJ was assayed in these different cell lines as described above; compared to parental MCF10A cells and to MCF10A/pBABE control cells, TRAF4 overexpression resulted in a 2-fold decrease in TJ-harboring cells (Figure 1C,E). To further substantiate the role of TRAF4 on TJs, we restored TRAF4 expression in MCF10A/shT4 cells (MCF10A/shT4+TRAF4) using an expression system insensitive to shT4-mediated down-regulation. TRAF4 expression was efficiently restored as the protein was expressed over six times higher than in parental cells (Figure 1A). MCF10A/shT4 cells transduced with the empty vector (pBABE) served as a negative control (MCF10A/shT4+pBABE). These cell lines were then assayed for the formation of TJs using ZO-1 staining on confluent monolayers. Similarly to TRAF4-silenced MCF10A cells, in the absence of rescue (MCF10A/shT4+pBABE), the number of TJs was two times higher than in parental and nonsilenced cells (Figure 1D,E). Remarkably, compared to parental MCF10A cells, rescued cells (MCF10A/shT4+TRAF4) had a 2-fold decrease in TJs similar to TRAF4 overexpressing cells (MCF10A/TRAF4) (Figure 1D,E). Thus restoring TRAF4 expression rescues the TJ phenotype induced by its silencing.

To study whether TRAF4 might regulate the expression level of proteins involved in TJs, adherens junctions, and desmosomes, levels of ZO-1, E-cadherin, beta-catenin, and desmoplakin were measured in the different cell lines by immunoblotting. Modulating TRAF4 expression did not modify significantly ZO-1, E-cadherin, beta-catenin, and desmoplakin protein levels (Figure S1A). In addition, immunofluorescence of endogenous beta-catenin showed that TRAF4 does not affect the membrane-bound beta-catenin pool, suggesting that TRAF4 primarily targets TJs (Figure S1B–D).

To know whether TRAF4 acts on TJ assembly, we next used the “calcium switch” model in MCF7 cells. This assay involves reversible disruption of epithelial junctions by extracellular calcium removal followed by a rapid reassembly triggered by calcium repletion [33]. MCF7 cells form well-defined TJs in tissue culture conditions [34] and endogenous TRAF4 is predominantly localized at TJs [17]. To address the role of TRAF4 on TJ assembly, we generated a TRAF4-silenced cell line in MCF7 cells using a shRNA strategy. Compared with parental (MCF7) and control (MCF7/shCtrl) cells, TRAF4 silencing (MCF7/shT4) resulted in a 90% reduction in protein levels (Figure S2A). We next examined if TRAF4 silencing affected reassembly of MCF7 TJs using the “calcium switch” assay. TRAF4 down-regulation accelerated the reassembly of TJs, as shown by the appearance of continuous junctional labeling of ZO-1 (Figure S2B). After 3, 5, and 7 h of calcium repletion, TRAF4-silenced cells showed more formed TJs than parental and control cells (Figure S2B,C). After 20 h of calcium repletion, all cells recovered TJs (Figure S2B). Moreover, reintroduction of TRAF4 in silenced cells rescued the phenotype of TRAF4-depleted cells, since cells expressing a sh-insensitive TRAF4 construct (MCF7/shT4+TRAF4) reassembled TJs in a kinetic comparable to that of parental MCF7 cells (Figure S2B,C).

Thus, in the normal MEC MCF10A, TRAF4 negatively regulates TJs. Moreover, in the malignant MEC MCF7 forming well-defined TJs, TRAF4 delays the reassembly of TJs. Collectively these data indicate that TRAF4 modulates TJs by delaying their formation and/or by favoring the dissociation of TJ.

### TRAF4 Is a Novel Phosphoinositide Binding Protein

**Figure 2:**
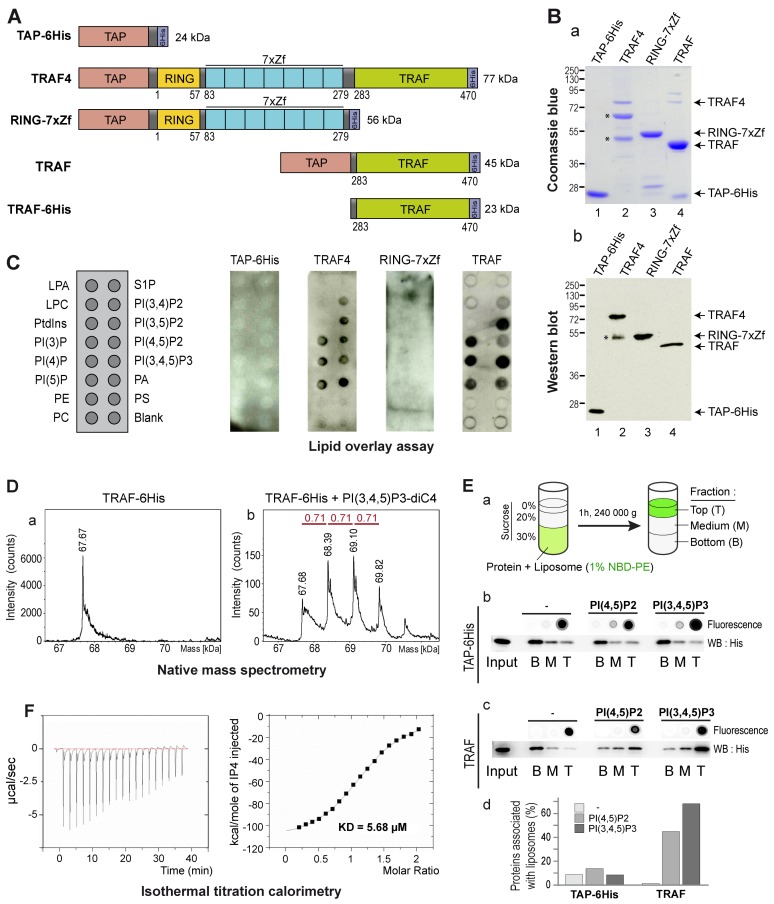
TRAF4 binds PIPs through its TRAF domain. (A) Schematic representation of the recombinant proteins used in lipid binding assays. TAP and 6His tags were used for the purification. RING, Zf, and TRAF are conserved structural domains present in the TRAF4 protein. (B) Coomassie blue staining (a) and Western blot analysis (b) of purified recombinant proteins. The antibody used recognized the immunoglobulin-binding domain of protein A from the TAP tag. TRAF4 degradation products are indicated by asterisks. (C) Lipid-overlay assay. Left, schematic view of a PIP-strip membrane. LPA, lysophosphatidic acid; S1P, Sphingosine-1-phosphate; LPC, Lysophosphocholine; PI, Phosphatidylinositol; PI(3)P, PI-(3)-phosphate; PI(4)P, PI-(4)-phosphate; PI(5)P, PI-(5)-phosphate; PI(3,4)P2, PI-(3,4-)bisphosphate; PI(3,5)P2, PI-(3,5)-bisphosphate; PI(4,5)P2, PI-(4,5)-bisphosphate; PIP(3,4,5)P3, PI-(3,4,5)-trisphosphate; PA, Phosphatidic acid; PE, Phosphatidylethanolamine; PS, Phosphatidylserine; PC, Phosphatidylcholine. The TAP-6His recombinant protein served as a negative control. Immunodetection of bound proteins was performed using a TAP-identifying antibody. TAP-6His and RING-7xZf did not bind to any membrane-coated lipids, while both full-length TRAF4 and the TRAF domain in isolation interacted with all PIPs and PA. (D) The TRAF domain of TRAF4 binds PIP in solution. Electrospray ionization time-of-flight mass spectrometry deconvoluted spectra of the TRAF domain of TRAF4 in the absence (a) and in the presence (b) of PI(3,4,5)P3-diC4. In isolation and in the absence of lipid, the TRAF domain is a trimer (a). In the presence of PIP, three additional peaks corresponding to one to three bound lipids are detected (b). Theoretical masses of the TRAF trimer and PI(3,4,5)P3-diC4 are 67.678 kDa and 0.714 kDa, respectively. (E) Liposomes flotation assay. a, schematic representation of the liposome flotation assay. Blank liposomes, PI(4,5)P2-containing liposomes, and PI(3,4,5)P3-containing liposomes were incubated with recombinant proteins, and liposome/protein-mixed fractions were separated by sucrose gradient ultracentrifugation. Binding of recombinant control TAP-6HIS (b) and TRAF domain of TRAF4 (c) to liposomes using membrane flotation assay. Fluorescent analyses (dot blot) of NBD-PE indicated that blank and PIP-containing liposomes were present in the top fraction. The presence of recombinant proteins in each fraction was detected by Western blot using anti-His antibody and quantified by densitometry-analysis using ImageJ software. The control TAP-6His was predominantly detected in the bottom fraction (b). In contrast the centrifugation profile of the TRAF4-TRAF domain was modified in the presence of PIP-containing liposomes (c). Indeed when mixed with blank liposomes, the TRAF domain was present in the bottom fraction, while in the presence of PI(4,5)P2) and PI(3,4,5)P3-containing liposomes, the TRAF domain was present in the top fraction. (F) Affinity of TRAF4 for PIP was measured by ITC. Titration was performed with 16 µM TRAF-6His recombinant protein, to which 500 µM of inositol-(1,3,4,5)-tetrakisphosphate were added incrementally. The TRAF domain of TRAF4 binds IP4 with a K_D_ of 5.68 µM.

In MECs, TRAF4 was shown to be present at the plasma membrane in a highly dynamic manner. Indeed fluorescent recovery after photobleaching (FRAP) experiments showed that the protein has a short residency time in the membrane. These experiments supported the notion that TRAF4 is shuttling between the plasma membrane, the cytoplasm, and possibly the nucleus [17]. In addition, in flies, TRAF4 was shown to interact with proteins from TJs, including PAR3 (Partitioning-defective 3) [21], making this protein a good candidate to explain TRAF4 addressing in junctions. However, in human cells, we failed to find a direct interaction between TRAF4 and PAR3 (F.A. unpublished data), suggesting that another mechanism is responsible for TRAF4 membrane targeting. Of interest, it was shown that adaptor proteins from TJs, including PAR3 and ZO-1, were localized to cell membranes via an interaction with membrane lipids belonging to the phosphoinositide (PIP) family [35],[36]. We thus reasoned that TRAF4 might be targeted to the cell membrane and possibly TJ by using a similar mechanism. To address the potential TRAF4 interaction with membrane lipids, we used an *in vitro* lipid binding assay called lipid overlay assay [37]. To this aim, recombinant TRAF4 protein was produced and purified from *E. coli*. Recombinant TRAF4 was flanked by two tags, a Tandem Affinity Purification (TAP)-tag [38] and a 6His-tag at the amino- and carboxy-terminal parts, respectively (Figure 2A). The efficiency of the purification was probed by Coomassie blue staining (Figure 2Ba) and Western blot (Figure 2Bb). Recombinant proteins were detected using an antibody recognizing the immunoglobulin-binding domain of protein-A from the TAP tag. We next tested the direct interaction of recombinant TRAF4 with lipids immobilized on membranes by lipid overlay assay (Figure 2C). While the control TAP-6His protein did not bind to any lipid, recombinant TRAF4 did bind to all PIPs and phosphatidic acid (PA). Interestingly, TRAF4 did not bind to other negatively charged lipids like phosphatidylserine and phosphatidylinositol (Figure 2C). Thus, lipid overlay assay showed that TRAF4 binds PIPs *in vitro*.

TRAF4 is a modular protein composed of a RING domain, seven TRAF-type zinc-fingers, and a TRAF domain (Figure 2A) [24]. To narrow down the domain involved in this binding, we produced two deletion mutants of TRAF4: one lacking the TRAF domain and thus only composed of the RING domain and of the seven zinc fingers (RING-7xZf) and the second one lacking all domains except the TRAF domain (TRAF) (Figure 2A). While the RING-7xZf part of the protein showed no detectable binding to lipids, the TRAF domain showed a lipid-binding profile similar to the wild-type TRAF4 protein (Figure 2C). This result shows that the TRAF domain of TRAF4 is responsible for the interaction of the protein with PIPs.

We next used a different method called native mass spectrometry to show the binding of the TRAF domain with PIPs. This method is sensitive enough to measure the molecular weight of lipid–protein complexes and address their stoichiometry [39]. In this assay, the recombinant TRAF domain in isolation (Figure 2A) was incubated with a soluble form of PI(3,4,5)P3 and analyzed by Electrospray Ionisation Time of Flight (ESI-TOF) mass spectrometry (Figure 2D). In absence of lipid, the TRAF domain fused to a 6His tag (TRAF-6His) was detected as a single peak of 67.7 kDa (Figure 2Da). Consistent with the trimerization property of the TRAF proteins via the TRAF domain [40], this peak represents three times the size of the monomeric TRAF domain (22.6 kDa). When TRAF-6His was incubated with PI(3,4,5)P3 prior to the ESI-TOF analysis, three additional major peaks were detected (Figure 2Db). Interestingly, each new peak has a size shift of ∼710 Da, which corresponds to the theoretical mass of one PI(3,4,5)P3 molecule. This indicates that the TRAF domain as a trimer can directly interact with one to three PI(3,4,5)P3 molecules (Figure 2D). Altogether, this shows that the recombinant TRAF domain of TRAF4 is well folded and trimerizes in solution similarly to the TRAF domains of TRAF2 and TRAF6 [40],[41]. In addition, it can bind up to three PI(3,4,5)P3 molecules, thus suggesting that each TRAF monomer has a binding site for one PIP molecule.

In addition, the ability of the TRAF domain to interact with PIPs was measured by direct binding to 100-nm large unilamellar vesicles (LUVs) by flotation in a sucrose gradient (Figure 2Ea) [42],[43]. All liposome preparations were labeled with a fluorescent lipid (NBD-PE), which allows for their direct visualization. Three different liposome preparations were tested: blank (no PIP), PI(4,5)P2-, and PI(3,4,5)P3-containing LUVs. Liposomes were first incubated with recombinant proteins, then mixed with sucrose, and finally allowed to float over this sucrose cushion in virtue of their lower density. After ultracentrifugation, liposomes followed by NBD-PE fluorescence were found in the top fraction of the gradient (Figure 2Eb,c). The control TAP-6His protein was never associated with liposomes (Figure 2Eb,d). Of interest, in presence of blank liposomes, the TRAF domain of TRAF4 was not associated with liposomes and was mainly present in the bottom fraction of the gradient (Figure 2Ec). In contrast, when mixed with PI(4,5)P2 or PI(3,4,5)P3-containing liposomes, the TRAF domain of TRAF4 was predominantly present in the top fraction in association with liposomes (Figure 2Ec,d). These data show that the TRAF domain of TRAF4 is able to interact with PIPs in the context of a biological membrane.

To gain insight about the affinity between the TRAF domain and PIPs, we performed isothermal titration calorimetry (ITC) experiments [44]. ITC was done with 16 µM TRAF-6His to which 500 µM inositol-(1,3,4,5,)-tetrakisphosphate (IP4) were added incrementally (Figure 2F). In these conditions, the TRAF domain is a homotrimer and the calculated numbers of IP4 binding sites indicated a 3∶1 stoichiometry of IP4 to TRAF-6His trimer. The lipid binding affinity was then calculated. The dissociation constant (K_D_) for one lipid-binding site of the TRAF domain and one IP4 molecule was 5.68 µM. This magnitude is consistent with K_D_ found for other PIP interacting proteins from TJs including PAR3 (K_D_ = 8 µM), ZO-1 (K_D_ = 1.3 µM), and ZO-2 (K_D_ = 2.6 µM) [35],[36].

Altogether, these data show that TRAF4 has the ability to bind to PIPs and PA. The protein exists as a homotrimer that binds up to three lipid molecules. This interaction is mediated by the TRAF domain and the affinity of binding is in the micromolar range, which is consistent with the K_D_ of other PIP-binding domains [36],[45],[46].

### The TRAF Domain Is a Novel *Bona Fide* PIP-Interacting Domain

**Figure 3:**
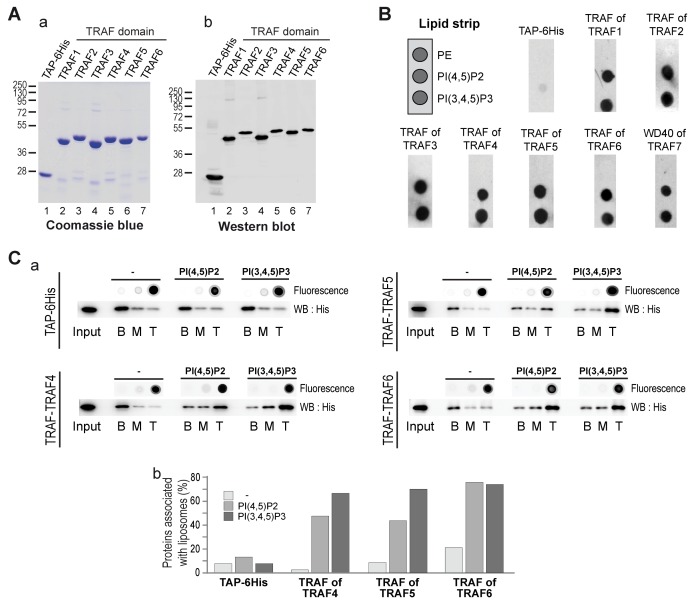
The PIP-binding ability is conserved through the TRAF family. (A) Coomassie blue staining (a) and Western blot analysis (b) of purified recombinant TRAF domains from the TRAF family. The antibody used recognized the immunoglobulin-binding domain of protein A from the TAP tag. (B) Lipid-overlay assay of TRAF domains from the TRAF family. Left, schematic view of a simplified PIP-strip. In this assay, the TAP-6His and the TRAF of TRAF4 are used as negative and positive control, respectively. Immunodetection of membrane-bound proteins was performed as described in Figure 2C. All TRAF domains from the TRAF family bind to PIPs. (C) (a) Binding of recombinant TRAF domains of TRAF4, TRAF5, and TRAF6 to liposomes was analyzed using liposome flotation assay as in Figure 2E. The control TAP-6His is unable to float in the presence of control and PIP-containing liposomes. In contrast, recombinant TRAF domains of TRAF5 and TRAF6 floated specifically when bound to PIP-enriched liposomes. (b) The quantification of proteins present in the different fractions was performed by Western blot and densitometry using ImageJ software.

Given that the TRAF domain is well conserved within the TRAF protein family, we reasoned that the other TRAF proteins might bind PIPs as well. To test this hypothesis, we produced and purified the TRAF domains of the five other human TRAF proteins (TRAF1 to 6) in fusion with a TAP and a 6His-tag. As described before, the purification of these different recombinant domains was probed by Coomassie blue staining (Figure 3Aa) and Western blot (Figure 3Ab), and they were tested using a simplified lipid overlay assay containing a negative control (PE) and the major plasma membrane localized PIPs, PI(4,5)P2 and PI(3,4,5)P3. Similar to previous results, the TAP-6His negative control did not bind to any lipids. Interestingly, the TRAF domains of the other TRAF paralogs (TRAF1 to TRAF6) interacted with PI(4,5)P2 and PI(3,4,5)P3 (Figure 3B). To support this finding, we performed liposome flotation assays with TRAF5 and TRAF6 TRAF domains. Consistent with lipid overlay assays, the TRAF domains of TRAF5 and TRAF6 were associated with PI(4,5)P2- and PI(3,4,5)P3-containing liposomes and not with blank liposomes (Figure 3C). This finding indicates that the TRAF domains of TRAF5 and TRAF6 have the ability to interact with PIPs in the context of a biological membrane. Interestingly, using lipid overlay we also found that the TRAF domain of the fly TRAF4, dTRAF1, interacts with PIPs (Figure S3).

Together, these experiments show that the TRAF domain is a *bona fide* PIP-interacting protein domain. They provide a novel link between the signaling adaptor proteins from the TRAF family and lipids.

### Structural Insight on PIP–TRAF Domain Interaction

**Figure 4:**
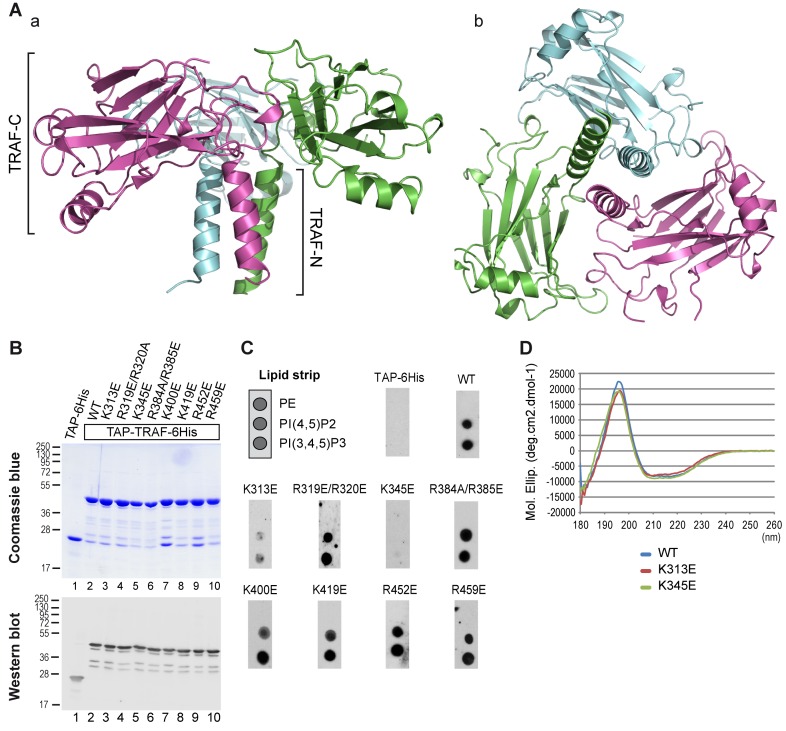
TRAF4-PIP binding involves lysines K313 and K345. (A) Ribbon drawing of the mushroom-shaped trimeric TRAF domain of human TRAF4. Three-fold axis vertical and into the page are shown in (a) and (b), respectively. The three TRAF monomers are colored in magenta, cyan, and green, respectively. The β-sheet regions (TRAF-C) exhibited proper three-fold symmetry. (B) Coomassie staining (upper panel) and Western blot analysis (bottom panel) of recombinant purified WT and mutant TRAF domains of TRAF4. Mutated residues are indicated on the top. The antibody used recognized the immunoglobulin-binding domain of protein A from the TAP tag. (C) Lipid-overlay assay of TRAF4 TRAF domain mutants. Left, schematic view of a simplified PIP strip. In this assay, the TAP-6His tag and the WT TRAF domain are used as a negative and positive control, respectively. Immunodetection of membrane-bound proteins was performed as described in Figure 2C. Mutagenesis of conserved basic residues showed that replacement of lysines 313 and 345 by a glutamic acid decreases and abolishes binding to PIPs, respectively. (D) Structural integrities of WT and mutant TRAF domains of TRAF4 were analyzed by circular dichroism. Replacement of lysines 313 and 345 with a glutamic acid did not affect the secondary structure of the corresponding mutant TRAF domains.

To get mechanistic insights about the interaction between the TRAF domain and PIPs, we determined the crystal structure of the TRAF domain of human TRAF4 (PDB 3ZJB). The structure was resolved to 1.85 Å by molecular replacement using the structure of human TRAF2 (PDB ID 1CA9; [40]) (Table S1). The structure was refined to convergence (R_work_ = 0.1632, R_free_ = 0.1995) and includes residues 283–470 of the TRAF domain (Figure 4A). No evidence was seen, however, for the presence of IP4 in the electron density maps. The most striking structural feature of the TRAF domain is the formation of a mushroom-shaped trimer with the coiled-coil domain (TRAF-N) as the stalk and the TRAF-C domain as the cap (Figure 4A), which is similar to the described structure of the TRAF domain of TRAF2 [40] and TRAF5 [47]. The structural architecture of the TRAF-C domain contains an eight-stranded antiparallel β-sandwich and a three turn helix present in the crossover connection between two β-strands as previously described for TRAF2, TRAF3, and TRAF6 proteins [48].

The structure of the TRAF domain was used to further characterize structural determinants involved in the binding with PIPs and to seek out TRAF4 mutants defective in this binding. Several protein domains including PH (pleckstrin homology), PX (phox homology), ENTH (Epsin N-terminal homology), FYVE (Fab1, YotB, Vac1p, and EEA1), and PDZ (PSD95, Dlg1, and ZO-1) domains bind PIPs [49]. These evolutionarily unrelated domains have in common the presence of at least two positively charged residues, lysine and/or arginine, directly interacting with PIPs [50]. Eleven lysines and 15 arginines are present within the TRAF domain of TRAF4. To identify critical residues for PIP binding within TRAF4, we focused on positively charged residues present at the surface of the TRAF domain (Figure S4). Eight positively charged residues—K313, R319/320, K345, R384/R385, K400, K419, R452, and R459 (Figure S4)—were selected and mutated independently into glutamic acid to produce and purify the corresponding recombinant proteins in *E. coli* (Figure 4B). These mutants were then tested by lipid overlay assay using a simplified lipid-coated membrane (Figure 4C). While six out of the eight mutants still bound PIPs (Figure 4C), the K313E and K345E TRAF4 mutants bound poorly and not at all to PIPs, respectively (Figure 4C). To exclude the possibility that the loss of PIP binding of both mutants was due to structural alterations, we checked their folding by circular dichroism spectroscopy, a method allowing the determination of protein secondary structures [51]. The near far-UV CD spectra of the two mutants were highly similar to that of the wild-type (WT) TRAF domain (Figure 4D), indicating that the K313E and K345E mutations did not affect the TRAF domain secondary structure. We also verified that the overall structure of the K345E mutant was unaffected using gel filtration and dynamic light scattering and showed that this mutant had an organization similar to the WT protein corresponding to a soluble trimer (Figure S5). This mutagenesis study showed that two positive amino acids, lysine 313 and lysine 345, are contributing and essential residues for the binding of TRAF4 to PIPs, respectively.

**Figure 5:**
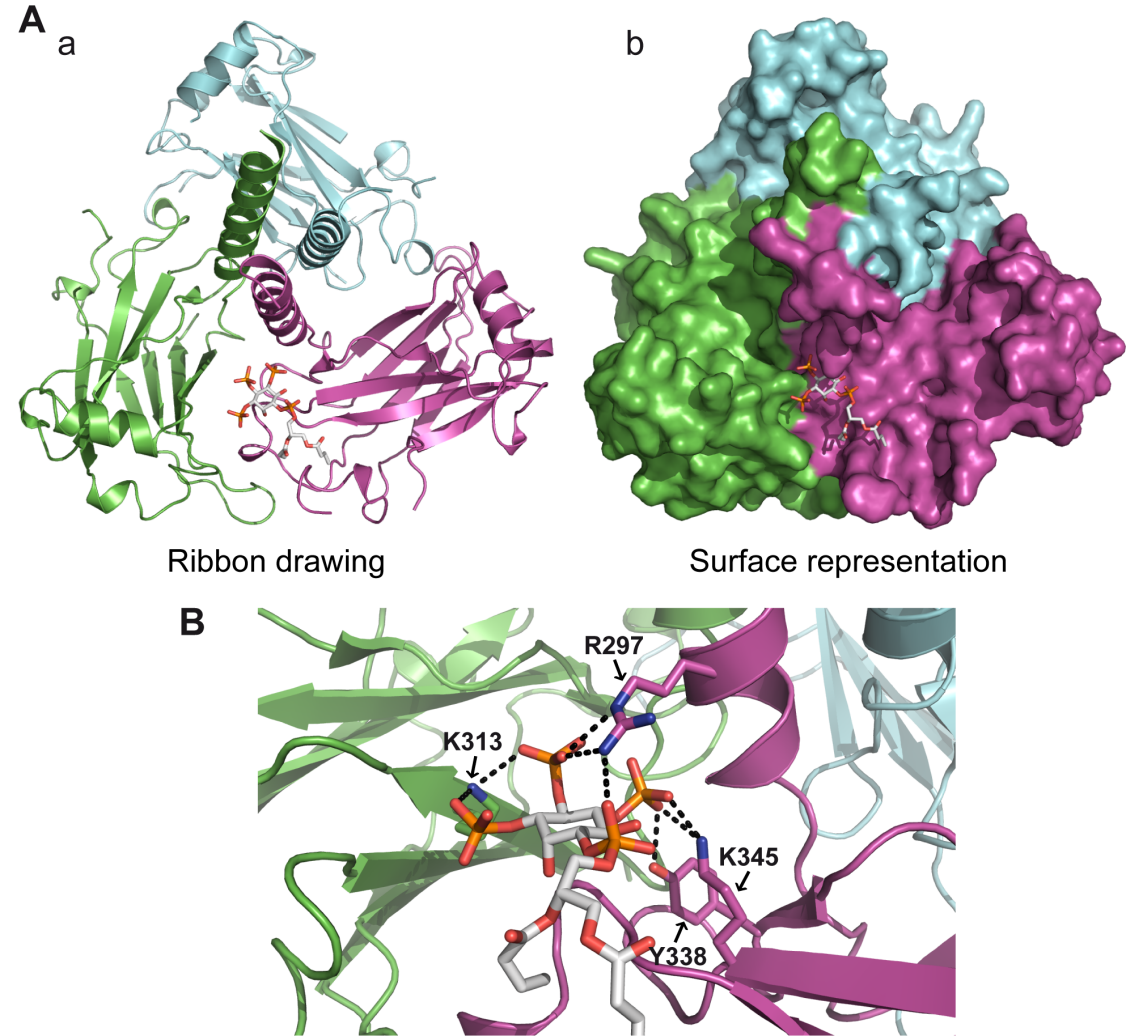
Modeling of the PIP3-diC4 binding onto the TRAF domain of TRAF4. (A) Model of PIP3-diC4 binding onto the TRAF domain of TRAF4 in ribbon drawing (a) and surface (b) representations. The PIP3-diC4 is bound in a pocket at the interface between two different TRAF monomers. (B) PIP3-diC4-interacting residues and PIP3-diC4 ligand are depicted in stick models. Phosphate, nitrogen, and oxygen atoms are colored in orange, blue, and red, respectively. Hydrogen bond interactions are shown as dashed lines. In this model, three basic residues (R297, K313, and K345) and one aromatic residue (Y338) interact directly with the lipid.

We next used this mutant analysis to build a model representing the binding of the fully deprotonated PI(3,4,5)P3-diC4 to the TRAF domain of TRAF4 (Figure 5). The GOLD program was used; indeed, this software is an automated ligand docking program broadly used to model ligand–protein binding [52]. Hydrogen bonding of the ligand to lysines 313 and 345 atoms was set as a prerequisite. In the model, which was further refined by energy minimization of the fully hydrated protein–ligand complex, the PI(3,4,5)P3-diC4 molecule binds to the TRAF domain at the interface between two protomers (Figure 5). Indeed, the lipid interacts with lysine 313 from one protomer and lysine 345 from the adjacent protomer. Lysine 313 directly interacts with both phosphates at positions 3 and 4 of the PI(3,4,5)P3-diC4, whereas lysine 345 only binds the phosphate at position 5 (Figure 5B). This model highlighted the presence of two other interacting residues, arginine 297, which interacts with the phosphate at position 4, and tyrosine 338, which binds to the phosphate at position 5. Interestingly, it has been reported in the literature that PIP-binding domains must have an aromatic residue (Tyr or His) that interacts with the lipid in addition to basic residues [50]. Altogether, these results show that TRAF4 is a novel PIP-binding protein that uses the TRAF domain, a mushroom-shaped trimer fold, to bind up to three lipid molecules.

### The TRAF Domain of TRAF4 Is Recruited at the Plasma Membrane Via Its Interaction with PIPs

**Figure 6:**
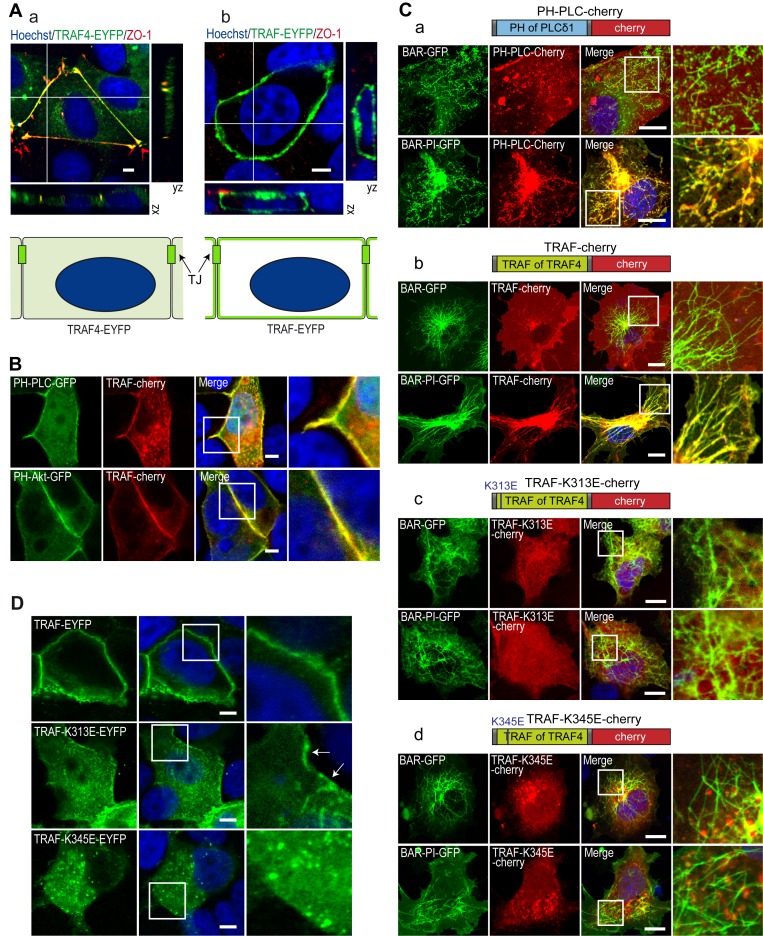
K313 is contributing and K345 is essential to the plasma membrane recruitment of the TRAF domain. (A) Colocalization of the complete TRAF4 protein and of the TRAF domain in isolation with the TJ protein ZO-1 in MCF7 cells. Cells transiently transfected with YFP-tagged (green) complete TRAF4 protein (a) and TRAF domain (b) were labeled for endogenous ZO-1 (red) and DNA (nuclei in blue). Confocal sections are shown together with xz- and yz-scans. Schematic representation of the localization of the EYFP-tagged proteins is presented at the bottom. TRAF-EYFP exhibited recruitment all along the plasma membrane, whereas TRAF4-EYFP is specifically targeted to the TJs. (B) Colocalization between the TRAF4-TRAF domain and fluorescently tagged PIP-probes. MCF7 cells were co-transfected with the mCherry-tagged TRAF domain and EGFP-tagged PH domains of the phospholipase C protein (PH-PLC-GFP, top) and Akt (PH-Akt-GFP, bottom). Confocal sections showed that the TRAF domain of TRAF4 co-localized with PH-PLC-GFP and PH-Akt-GFP proteins that bind PI(4,5)P2 and PI(3,4,5)P3 at the apical and basolateral side of the cell, respectively. Nuclei were stained using Hoechst (blue). Insets on the right represent a 2.5× magnification. Scale bar, 5 µm. (C) Tubulation assays in COS7 cells. The recruitment of PH-PLC-Cherry (a), WT TRAF-Cherry (b), and mutant TRAF domains TRAF-K313E-Cherry (c) or TRAF-K345E-Cherry (d) on membrane tubes was studied by colocalization with BIN1/BAR-GFP (top panels) and BIN1/BAR-PI-GFP (bottom panels). Nuclei were stained using Hoechst (blue). Similarly to PH-PLC-Cherry, a known PI(4,5)P2 binding domain, TRAF-Cherry protein was specifically recruited to PIP-enriched membrane tubes. Both lysines 313 (c) and 345 (d) mutations prevent the recruitment of the TRAF domain to PIP-enriched membrane tubes. (a–d) Confocal sections; insets on the right are 3.5× magnification, Scale bar, 5 µm. (D) Confocal sections of MCF7 cells transfected with WT and mutant TRAF domains of TRAF4 fused to EYFP (green). The WT TRAF domain is recruited to the plasma membrane (top panels). While the K313E mutant (middle panel) is mostly cytoplasmic, a small fraction of the protein still localizes to the plasma membrane (arrows). The K345E mutant is only detected in the cytoplasm (bottom panel). Nuclei were stained using Hoechst (blue). Insets on the right are 3× magnification. Scale bar, 5 µm.

Even though they are quantitatively minor components of membranes, PIPs play a crucial role in cellular compartmentalization and in protein targeting [49],[53]. We hypothesized that owing to its affinity for PIPs, the TRAF domain would be targeted to PIP-enriched membranes. First, we compared the subcellular localization of the full-length TRAF4 protein to that of the TRAF domain in isolation in MCF7 MECs. To this aim, we expressed EYFP-tagged full-length TRAF4 or the TRAF domain of TRAF4 in MCF7 and labeled them with the TJ marker ZO-1 (Figure 6A). Consistent with a previous report [17], TRAF4 is mainly localized in TJs (Figure 6Aa). Strikingly, in isolation, the TRAF domain was distributed homogenously along the plasma membrane and was not enriched in TJs (Figure 6Ab). A variety of PIP species can be found along cellular membranes. We next looked at whether the localization of specific PIPs could explain the recruitment of the TRAF4-TRAF domain all along the plasma membrane in MCF7 cells. Two major plasma membrane PIPs, PI(4,5)P2 and PI(3,4,5)P3, can be localized using specific probes, the GFP-tagged PH domain of phospholipase Cδ (PH-PLCδ) and the GFP-tagged PH domain of Akt (PH-Akt), respectively [54],[55]. PI(4,5)P2 and PI(3,4,5)P3 mark the apical and basolateral membranes, respectively [56],[57]. We therefore analyzed the localization of the TRAF domain with respect to the subcellular distribution of PI(4,5)P2 and PI(3,4,5)P3 in MCF7 cells. Both the PH-PLCδ and the PH-Akt probes colocalized with the TRAF-Cherry protein (Figure 6B), indicating that in isolation the TRAF domain of TRAF4 is localized in both PI(4,5)P2- and PI(3,4,5)P3-enriched regions that include most of the plasma membrane.

The co-localization of the TRAF domain with the major plasma membrane PIPs suggests that it is recruited to membranes owing to its interaction with PIPs. To test this hypothesis, we performed a BAR (Bin-Amphiphysin-Rvs) domain-induced membrane tubulation assay [58]. In this assay, membrane tubes are induced in the cytoplasm of COS-7 cells by the expression of the BAR domain of BIN1 (Figure 6C) [58]. Moreover, by using the isolated BAR domain and a fusion between the BAR and the PI domains of BIN1 protein, one can initiate the formation of numerous intracytoplasmic naked- and PI(4)P/PI(4,5)P2-decorated membrane tubes, respectively. To calibrate this assay, we first studied the recruitment of the known PI(4,5)P2 interactor PH-PLCδ in both naked- and PIP-decorated BAR-induced membrane tubes (Figure 6Ca). Consistent with the known affinity of this sensor for PI(4,5)P2 [59], the mCherry-tagged PH-PLCδ protein hardly co-localized with naked tubes induced by the BIN1/BAR construct; conversely, in cells harboring PIP-decorated membrane tubes induced by the BIN1/BAR-PI construct, the mCherry-tagged PH-PLCδ was massively recruited onto membrane tubes (Figure 6Ca). We next studied the recruitment of the mCherry-tagged TRAF domain in this system (Figure 6Cb). Remarkably, the TRAF domain was massively recruited onto PIP-decorated membrane tubes (Figure 6Cb, bottom panel) and did not colocalize with naked tubes (Figure 6Cb, upper panel). This result shows that the TRAF domain is specifically recruited onto PIP-enriched membranes and supports the notion that this interaction is necessary for its subcellular localization.

We then used the BAR domain-induced membrane tubulation assay to study the membrane recruitment of PIP-binding–deficient TRAF mutants, which have been previously characterized biochemically (Figure 4). To this aim, the K313E and K345E mutants were constructed in fusion with the mCherry protein (Figure 6C) and expressed in COS-7 cells in the presence of either BIN1/BAR- or BIN1/BAR-PI-induced membrane tubes. In contrast to the WT TRAF domain, which colocalizes specifically with PIP-decorated tubes (Figure 6Cb), both mutants did not colocalize with either naked- or PIP-decorated membrane tubes and were cytoplasmic (Figure 6Cc–d).

We also looked at the subcellular localization of these two PIP-binding–deficient mutants in MCF7 cells. In these cells the WT TRAF domain is localized at the plasma membrane (Figure 6D, upper panel). Consistent with the biochemical *in vitro* binding assays that showed that the K313 and the K345 residues were contributive and essential to the binding with PIPs, respectively (Figure 4), the TRAF-K313E mutant was localized both in the cytoplasm and at the plasma membrane (Figure 6D, middle panel, arrows), while the TRAF-K345E mutant was completely absent from the plasma membrane and exclusively present in the cytoplasm (Figure 6D, bottom panel). Altogether, these data are consistent with the notion that the subcellular localization of TRAF4 at the plasma membrane is governed by its binding to membrane resident PIPs.

### TRAF–PIP Interaction Is Crucial for TRAF4 Recruitment in TJs

**Figure 7:**
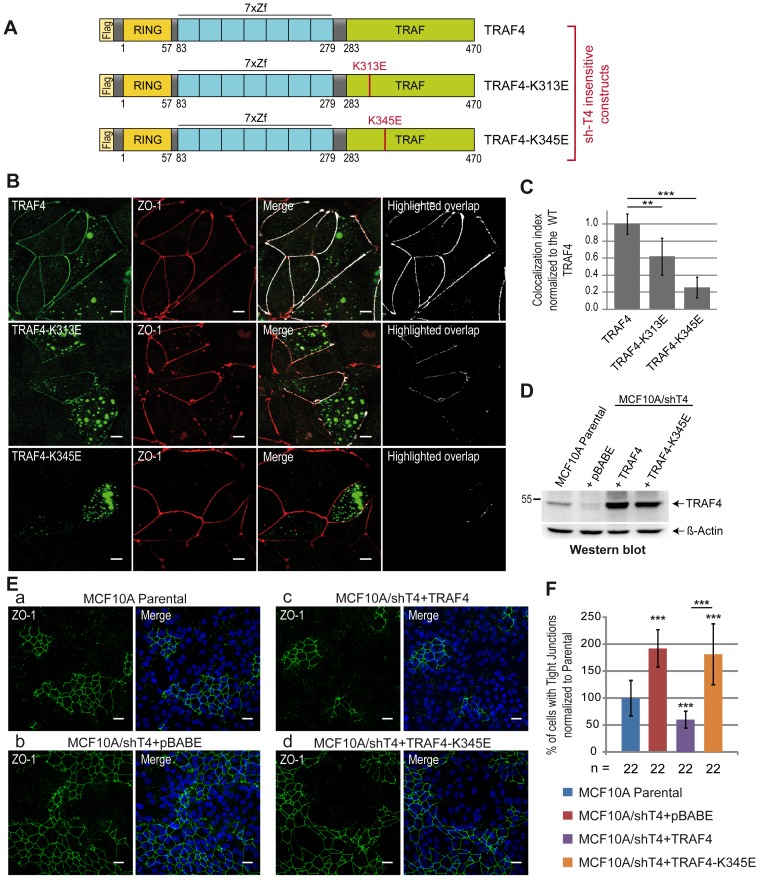
TJ recruitment of TRAF4 is PIP-binding dependent. (A) Schematic representation of sh-insensitive Flag-tagged WT and mutant TRAF4 constructs used to reintroduce TRAF4 expression in MCF7/shT4 cells. (B) Recruitment of WT and mutant TRAF4 proteins (green) at TJs was analyzed by colocalization with ZO-1 (red). The highlighted overlap (white) between TRAF4 and ZO-1 staining is shown on merge panels and alone on the right panel. While the TRAF4-K313E mutant is still partially colocalized with ZO-1 (middle panels), the TRAF4-K345E mutant does not colocalize anymore with ZO-1 (bottom panels). Scale bar, 10 µm. (C) Quantification of WT and mutant TRAF4 recruitment at TJs. The colocalization index (overlapping area between TRAF4 and ZO-1 staining divided by the TJ length) was measured on 10 microscopic fields. Compared to the WT protein, the colocalization index was reduced by 40% and 78% for K313E and K345E TRAF4 mutants, respectively. (D) Western blot analysis of TRAF4 protein level in parental MCF10A and in TRAF4-silenced cells (MCF10A/shT4) where WT (MCF10A/shT4+TRAF4) and mutant (MCF10A/shT4+TRAF4K345E) TRAF4 expression was restored. The MCF10A/shT4+pBABE cell line represents a control line transduced with the empty vector. Beta-actin was used as a loading control. (E) The presence of TJs was estimated by ZO-1 staining in parental (a) and in TRAF4-silenced cells where the expression of WT (c) and mutant TRAF4 (d) was reintroduced. TRAF4-silenced cell line transduced with the empty vector (b) was used as a control. The PIP-binding–deficient TRAF4-K345E cannot rescue the phenotype induced by TRAF4 silencing on TJs. Left panels, representative confocal image sections of ZO-1 staining (green); right panels, merge with Hoechst staining (blue). Scale bar, 20 µm. (F) TJ quantification in cell lines described in (D) and (E) was performed as described in Figure 1E. n, number of microscopic fields used for the quantification.

Compared with the complete protein that localizes predominantly in TJs, the TRAF domain of TRAF4 in isolation has a broader localization all along the plasma membrane. To know whether the binding with PIPs is a prerequisite for the addressing of the complete TRAF4 protein in TJs, we studied the subcellular localization of PIP-binding–deficient TRAF4 mutants in the context of the whole protein. To this aim, sh-insensitive Flag-tagged WT or mutant TRAF4 proteins (Figure 7A) were expressed in the MCF7/shT4 cell line (Figure S2). We used this experimental setting to avoid misinterpretations due to the possible trimerization of ectopically expressed TRAF4 with endogenous TRAF4 protein. The localization of the proteins to TJs was then evaluated by quantification of their colocalization with ZO-1 and the derivation of a colocalization index (Figure 7B). A complete colocalization between WT TRAF4 and ZO-1 was measured confirming its presence in TJs (Figure 7B). Consistent with the finding that the K313 residue contributes but is not essential to the binding of TRAF4 with PIPs, the TRAF4-K313E mutant only partially localized with ZO-1 (Figure 7B, middle panel). Interestingly, the TRAF4-K345E mutant was absent from TJs (Figure 7B). Indeed, after quantification, the colocalization index was reduced by 40% and 80% for the TRAF4-K313E and TRAF4-K345E mutants, respectively (Figure 7C). Taken together, these data show that PIP binding is a prerequisite for the localization of TRAF4 at TJs.

### TRAF4 Functions on TJs in a PIP-Binding–Dependent Manner

Our results indicate that TRAF4 is a negative regulator of TJs in MECs (Figure 1), and the TRAF domain is a novel PIP-binding domain that is essential for the addressing of the protein to the plasma membrane. In addition, we showed that the PIP-binding–dependent membrane recruitment of TRAF4 is necessary for its addressing to TJs. We then wondered whether the role of TRAF4 on TJs was dependent on its association with PIPs at the membrane and moreover on its presence to TJs. To address this, the K345E PIP-binding–deficient mutant of TRAF4 was used to rescue the TJ phenotype found in TRAF4-silenced MCF10A cells (MCF10A/shT4) (Figure 1). Both WT and mutant TRAF4 proteins were efficiently expressed in MCF10A/shT4 cells to levels above endogenous TRAF4 (Figure 7D). As previously mentioned (Figure 1B), TRAF4-silenced cells (MCF10A/shT4+pBABE) have a ∼2-fold increase in cells with TJs (Figure 7Ea and b,F), while cells with restored TRAF4 expression had 2-fold less TJs than the parental (Figure 7Ea and c,F). In contrast, restoring TRAF4 expression by using the TRAF4-K345E mutant did not rescue the phenotype induced by TRAF4 silencing (Figure 7Ec and d,F).

These results provide a novel illustration of the importance of TRAF4 subcellular localization on cell biology. Indeed, they show that to act as a negative regulator on TJ formation and/or stability, TRAF4 must be addressed to the plasma membrane/TJ via a PIP-binding–dependent mechanism. Therefore, preventing or favoring its presence in the plasma membrane and in TJs represents a means to regulate its action on tissue homeostasis.

### TRAF4 Promotes MEC Migration

**Figure 8:**
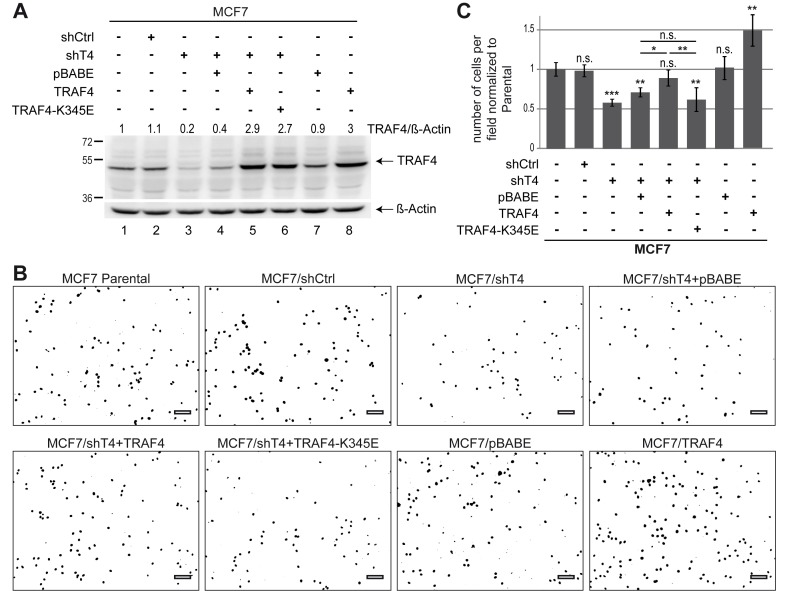
TRAF4 promotes MCF7 cell migration. (A) Western blot analysis of TRAF4 expression. In MCF7 cells, TRAF4 expression has been silenced (lanes 2–6), increased (lane 8), and restored in silenced cells using the WT (lane 5) and the K345E mutant (lane 6). Parental (lane 1), control shRNA (lane 2), and control expression vector (lane 7) together with a TRAF4 silenced line transduced with the empty vector (lane 4) were used as controls. TRAF4 expression levels were normalized to control parental cells using β-actin as loading control; values are indicated on the top. (B) Representative microscopic field of the bottom side of the transwell. Migrating cell nuclei were stained with Hoechst, and images are shown as inverted look-up table. (C) Bar chart representing the quantification of cell migration in MCF7 cells. The number of cells that migrated were counted and normalized to control parental cells. Thirty-six microscopic fields from three independent experiments were used for the quantification.

Of interest, TJ proteins, in addition to their characterized role in mediating cell–cell adhesion and assuring an epithelial barrier, can have an active role in cell migration. The protein ZO-1 actively regulates cell migration by modulating cytoskeletal dynamics [60]. Given that TRAF4 is overexpressed in breast cancer and that migration of cancer cells participates in tumor progression, we addressed the role of TRAF4 in the migration of breast cancer cells. To this aim, TRAF4 expression was modulated in MCF7 cells, and cell migration was measured using the Boyden chamber assay. We first studied cell migration in TRAF4-depleted MCF7 cells. Compared to parental (MCF7) and to a control cell line (MCF7/shCtrl), TRAF4 was expressed by less than 20% in TRAF4-silenced cells (MCF7/shT4) (Figure 8A). Migration was impaired by 40% in this line compared to the controls (Figure 8B,C). We next reintroduced TRAF4 expression in this silenced cell line (MCF7/ShT4+TRAF4), and TRAF4 was expressed at a level above the parental cells (Figure 8A). Consistently, reintroduction of TRAF4 was associated with increased cell migration similar to parental MCF7 cells (Figure 8B,C). To complement these findings, MCF7 cells stably expressing TRAF4 were generated (MCF7/TRAF4). TRAF4 was increased 3-fold as compared to parental and control cells (Figure 8A). Supporting the role of TRAF4 in cell migration, compared with parental and control lines, migration was increased by 40% in these cells (Figure 8B,C). To know whether this promigratory function is linked with its ability to interact with PIPs and to localize at TJs, we then used the PIP-binding–deficient TRAF4-K345E mutant in the Boyden chamber migration assay. To this end, this mutant was reintroduced and expressed in TRAF4-silenced cells (MCF7/shT4+TRAF4-K345E) (Figure 8A). In contrast to the WT protein, the TRAF4-K345E mutant did not restore cell migration in TRAF4-silenced MCF7 cells, indicating that a PIP-binding–deficient mutant could not rescue the migration phenotype induced by the loss of TRAF4 expression (Figure 8B,C). The positive impact of TRAF4 on cell migration was also addressed in MCF10A cells. Consistent with the results obtained using MCF7 cells, TRAF4 positively regulated cell migration in this cell line (Figure S6). Altogether, these results support the role of TRAF4 as a novel regulator of cell migration operating at the TJ level in a PIP-binding–dependent manner.

## Discussion

In the normal breast, TRAF4 is predominantly localized in TJs of polarized epithelial cells. The molecular mechanism targeting TRAF4 to these adhesion structures as well as its function at these regions remained elusive. The experiments presented here provide for the first time, to our knowledge, a molecular explanation for the recruitment of TRAF4 in TJs. Indeed TRAF4 uses its TRAF domain as a novel PIP-binding domain to be addressed to the plasma membrane, an essential step for its recruitment in TJs. Moreover, they show that TRAF4 acts as a negative regulator of TJs and favors migration of breast cancer cells.

Several lines of evidence indicate a link between TRAF4 and cell polarity [24]. During development, breast epithelial cells polarize and assemble into duct and acini structures. One of the earliest manifestations of breast cancer is the loss of this cellular organization. Indeed, loss of cell–cell contact and epithelial polarity are hallmarks of carcinomas and contribute to their development as carcinomas *in situ* or their progression to invasive adenocarcinomas. TRAF4 expression is altered in breast carcinomas and the protein shifts from TJs to other subcellular territories, suggesting that it could be part of the mechanisms leading to the disruption of the polarized breast epithelium.

Of interest, the epithelial polarity program relies on several conserved cellular machineries, including the domain-identity machinery that builds a TJ fence between apical and basolateral plasma membrane domains by using specific proteins and lipids [61]. The asymmetric distribution of lipids from the PIP family within the plasma membrane plays a key role in the establishment of cell polarity. These lipids represent optimal signaling mediators by forming docking sites for PIP-binding protein effectors [49],[54],[62]. To date, 11 PIP-binding domains have been described, including PH (pleckstrin homology), PX (Phox homology), and FYVE (Fab1, YOTB, Vac1, and EEA1) domains [63]. While the majority of PIP-binding modules selectively bind to one PIP depending on its phosphorylation status, it has recently emerged that some proteins bind PIPs in a promiscuous manner. It is the case for the polarity proteins PAR3, ZO-1, and α-syntrophin. They do not contain a consensus PIP-binding motif but use their PDZ or PH domains to bind PIPs [35],[36],[64]. It has been proposed that the loose binding of these proteins to PIPs served to enhance their affinity for membranes and that additional factors are required to fine-tune their subcellular localizations [49]. In this study, we show that TRAF4 uses its TRAF domain as a novel PIP-binding module. The TRAF domain is highly conserved within the family of TRAF proteins; consistent with this conservation we also show for the first time to our knowledge that the TRAF domains of TRAF1 to −6 also bind PIP. Therefore, the TRAF domain can be considered as a novel *bona fide* PIP-binding domain. Similarly to PAR3, ZO-1, and α-syntrophin, the TRAF domain of TRAF4 has a broad affinity for PIPs. Consistently, the TRAF domain in isolation localizes homogenously along the plasma membrane, while the full-length TRAF4 protein is restricted to TJs, suggesting that the binding with PIP is a prerequisite for the addressing of TRAF4 in TJs. Additional mechanisms are subsequently required to refine TRAF4 localization to TJs. Moreover, we solved the 3D structure of the TRAF domain of TRAF4 and showed that it exists as a trimer. Ligand-binding studies show that under its trimeric form TRAF4 can bind one to three lipid molecules, and thus avidity might increase its affinity to lipid membranes. These structural data suggest that the TRAF domain serves to enrich TRAF4 at the plasma membrane and its membrane association can be modulated by the local concentration in PIPs.

The TRAF4-TRAF trimer has a mushroom-shaped structure. The globular part defined as TRAF-C forms the cap, and the coiled-coil part known as TRAF-N is the stalk. To date, among the solved TRAF domains from TRAF2, TRAF5, and TRAF6 proteins [40],[65],[66], all share this specific architecture. In the canonical mode of action, TRAFs are cytoplasmic adaptor proteins that bind to the cytoplasmic tail of activated TNF and interleukin-1/Toll-like receptors to mediate a wide range of biological functions including immune and inflammatory responses [67]. When the TRAF domain of TRAF2 and TRAF6 were crystallized in the presence of a peptide representing their receptor ligand, the structure showed the peptide bound to a shallow surface depression on the side of one protomer without contact to the adjacent protomer [40],[66]. This mode of binding is very distinct from the PIP-binding model that we predict from the structure of the TRAF domain. In this model, each PIP molecule binds at the interface between two neighboring protomers. Very interestingly, the superimposition of the receptor peptide tail and lipid binding sites shows that they do not overlap. Therefore, a synergistic interaction between both modes of binding is possible in theory. The measured affinity constants between TRAF proteins and their receptor ligand were quite low (40–60 µM range) [44],[68], and it has been proposed that the high and specific affinity for TRAF proteins with their receptors is achieved through avidity [48]. Our results suggest that, in addition, a synergistic interaction of TRAF proteins with a membrane lipid would likewise stabilize and orient the TRAF adaptors at the membrane and increase interaction with their receptors [44],[68]. This potential mechanism remains to be addressed experimentally.

Other novel results from this study are the function of TRAF4 on TJ and cell migration. These findings provide a rationale for most of the developmental defects that were reported in TRAF4-deficient animals. For example, it has been shown that TRAF4 is essential for neural tube closure (NTC) and neural crest cell development, two processes involving TJ remodeling prior to cell migration [69],[70]. Indeed, TRAF4 knock-down in *Xenopus laevis* causes neural plate-folding defects and impairs neural crest cell formation, whereas TRAF4 overexpression expends the neural crest [12]. TRAF4 involvement in NTC has also been described in mice as TRAF4-deficient mice exhibit NTC defects giving rise to mild *spina bifida* phenotypes and embryonic lethality [13]. In addition, it has been recently shown that TRAF4 is a direct target of Twist, a transcription factor involved in neural crest formation and fate determination in frog [71]. Our results are consistent with these observations and suggest that TRAF4 contributes to TJ plasticity, a key process regulating NTC and neural crest cell formation and migration. Thus, we believe that in TRAF4 knock-out animals, neural crest cells might fail to disrupt their TJs, impairing their ability to undergo epithelial-to-mesenchymal transition and to achieve their proper destination contributing to the observed abnormalities. Cell–cell adhesion is an important regulator of cell migration [72]. In our experiment we did not notice significant alteration of molecular components involved in adherens junctions and in desmosomes. Nevertheless, we cannot rule out that these structures are altered by TRAF4 expression. Part of TRAF4 function might be linked with desmosomes and adherens junctions.

To conclude, TRAF4 acts both as a negative regulator of TJs and a cell migration promoter. To achieve its function, TRAF4 needs to interact with PIPs, which allows for its trafficking to the plasma membrane and subsequently to the TJs. This suggests that TRAF4 acts in a signaling loop involving lipids; however, the molecular mechanisms involved remain unclear and will be addressed in the future. Importantly, gain of TRAF4 expression and protein mislocalization have been reported in a variety of carcinoma. Notably, TRAF4 was found to be overexpressed through gene amplification in about 20% of breast cancers [1],[3],[73]. Interestingly, two recent reports showed that TRAF4 favors breast cancer cell migration [74],[75]. In particular, Zhang et al. showed that TRAF4 regulates the TGF-β pathway and that TRAF4 expression favors breast cancer metastasis and is associated with a poor prognosis among breast cancer patients [74]. Therefore, gain of TRAF4 function appears to be an important factor for the development and progression of breast cancer.

## Materials and Methods

### Cell Culture, Transfections, and Infections

COS7 cells were maintained in DMEM supplemented with 5% fetal calf serum (FCS) and 40 µg/ml gentamycin. MCF7 cells were grown in DMEM supplemented with 10% FCS, 0.6 µg/ml insulin, and 40 µg/ml gentamycin. MCF10A cells were cultured in DMEM/HAM F12(3∶1) supplemented with 20 µg/ml adenine, 5 µg/ml insulin, 5 µg/ml human apo-Transferrin, 1.5 ng/ml triiodothyronin, 2 ng/ml hEGF, 0.5 µg/ml hydrocortisone, 10% FCS, and 40 µg/ml gentamycin. Plasmid transfection was performed with Fugene6 transfection reagent (Roche) according to the manufacturer's protocol. For retroviral infection, retroviral vectors were co-transfected with pCL-Ampho vector (Imgenex) into a 293T retroviral packaging cell line using Fugene6 reagent. For lentiviral infection, pLKO.1 vectors were cotransfected with three packaging plasmids—pLP1, pLP2, and pLP/VSVG (Invitrogen)—into the 293T cell line using Fugene6 reagent. Both retroviral and lentiviral particles were collected 48 h after transfection, supplemented with 10 µg/ml polybrene and 20 mM Hepes, and incubated with MCF7 or MCF10A cells. Cells were then selected by addition of 0.5 mg/ml puromycin for lentiviral infection or 10 µg/ml blasticidin for retroviral infection.

### Cloning and Constructs

A pET28a(+)-TAP/6His expression vector was made by inserting a sequence encoding the TAP tag between the NdeI and NcoI restriction sites of the pET28a(+) vector (Novagen) [76]. To produce the recombinant protein corresponding to the TAP and the 6His tags in fusion, the synthetic oligonucleotide 5′-GGA TCC GAA TTC GTT AAC CTC GAG GCG GCC GC-3′ was cloned into the BamHI and NotI restriction sites of the pET28a(+)-TAP/6His vector. To produce recombinant proteins flanked by a TAP and a 6His tag at the amino- and carboxy-terminal extremity, respectively, the coding sequence of the full-length TRAF4 (pET28a(+)-TAP-TRAF4-6His), the RING-7xZf domains (pET28a(+)-TAP- RING-7xZf -6His), or the TRAF domain (pET28a(+)-TAP-TRAF-6His) of TRAF4 were amplified by PCR using the synthetic primers GAGA GGA TCC ATG GCG CCT GGC TTC/GAGA GTC GAC TCA GCT GAG GAT CTT CCG, GAGA GGA TCC ATG GCG CCT GGC TTC/GAGA GTC GAC TCA ACA CAT CAT GGC CAG, and GAGA GGA TCC GCC CTG GTG AGC CGG/GAGA GTC GAC TCA GCT GAG GAT CTT CCG, respectively. Sequences encoding TRAF1, TRAF2, TRAF3, TRAF5, and TRAF6 TRAF domains were amplified by RT-PCR from human liver RNA using the following primers: TRAF1, GAGA GGA TCC CAG ACC CTG GCC CAG AAA GA/GAGA GTC GAC AGT GCT GGT CTC CAC AAT GC; TRAF2, GAGA GGA TCC CAA GAC AAG ATT GAA GCC CT/GAGA GTC GAC GAG CCC TGT CAG GTA CAC AA; TRAF3, GAGA GGA TCC GGC CTG CTG GAG TCC CAG CTG AG/GAGA GTC GAC GGG ATC GGG CAG ATC CGA AGT AT; TRAF5, GAGA GGA TCC GCC GTT TTA GAA GAG GAA ACT A/GAGA GTC GAC GAG ATC CTC CAG GTC AGT TAA GT; TRAF6, GAGA GGA TCC CGC CTT GTA AGA CAA GAC CA/GAGA GTC GAC TAC CCC TGC ATC AGT ACT TC. The sequence encoding dTRAF1 was amplified by RT-PCR from S2 cells RNA using the following primers: GAGA GGA TCC GCC CTC AGC TCG CGC CAG GG/GAGA GTC GAC AAC GGC CAC TAT CTT GCT GG. PCR products were cloned into the BamHI and SalI restriction sites of the modified pET28a(+)-TAP/6His.

The sequence encoding the TRAF domain of TRAF4 was inserted between the NcoI and XhoI restriction sites of the pET28a(+) vector after PCR amplification using the synthetic primers AGA CCA TGG CCC TGG TGA GCC GGC AAC GG/AGA CTC GAG GCT GAG GAT CTT CCG GGG CAG to generate a vector encoding the 6His-tagged TRAF domain of TRAF4.

The pRK7N TRAF4 plasmid expressing Flag-tagged TRAF4, the pEYFP-N1-TRAF4, and pEYFP-N1-TRAF plasmids designed to express EYFP-fused TRAF4 and TRAF domain of TRAF4 in isolation, respectively, were previously described [77].

The BIN1/BAR and the BIN1/BAR-PI expression plasmids were kind gifts from Dr. Karim Hnia (IGBMC). The PH-PLCδ1-GFP and the PH-Akt-GFP expression plasmids were kind gifts from Dr. Bruno Beaumelle (Centre d'études d'agents Pathogènes et Biotechnologies pour la Santé, Montpellier, France) and Dr. Nicolas Vitale (Institut des Neurosciences Cellulaires et Intégratives, Strasbourg, France). The PH-PLCδ1 coding sequence was subcloned into the XhoI and SalI restriction sites of the pmCherry-C1 vector [78] after PCR amplification using the synthetic oligonucleotides GAG ACT CGA GCA ATG GAC TCG GGC CGG/GAG AGT CGA CTC ACT GGA TGT TGA G to generate pmCherry-C1-PH-PLCδ1 expression plasmid.

The sequence encoding the TRAF domain of TRAF4 was inserted between the KpnI and BamHI restriction sites of the pmCherry-N1 vector after PCR amplification using the synthetic primers GAG AAA GCT TGC GCC CTG GTG AGC CGG CAA CGG/GAG AGT CGA CTCA GCT GAG GAT CTT CCG GGG CAG to generate a vector encoding the TRAF domain of TRAF4 fused to pmCherry.

To obtain a shRNA expression vector targeting TRAF4 (target sequence: CCA GGA CAT TCG AAA GCG AAA) and a control vector (target sequence: CAA CAA GAT GAA GAG CAC CAA), annealed oligonucleotides were cloned into the pLKO.1 vector to generate pLKO.1-shT4 and pLKO.1-shCtrl vectors, respectively [79].

The retroviral pBABE vector was a kind gift from Dr. Pattabhiraman Shankaranarayanan (IGBMC). The sequence encoding TRAF4 was inserted into the EcoRI restriction site of the pBABE vector after PCR amplification using the synthetic primers GAG ACA ATT GCC CGC CAT GGC GCC TGG CTT CGA CTA CAA GTT C/GAG ACA ATT GTC AGC TGA GGA TCT TCC GGG G.

Site-directed mutagenesis was performed using the synthetic oligonucleotides: 5′-GTG CTC ATC TGG GAG ATT GGA TCC TAT GGA CGG CGG-3′, 5′-GGC AGC TAT GGA GCG GAG CTC CAG GAG GCC AAG-3′, 5′-AAG TAT GGT TAC GAG CTC CAG GTG TCT GCA-3′, 5′-GTG GCC CTT TGC TGC AGA AGT CAC CTT CTC C-3′, 5′-GAT CAG AGC GAC CCC GGG CTG GCT GAA CCA CAG CAC-3′, 5′-TGG AAG AAT TTC CAG GAA CCC GGG ACG TGG CGG GGC TCC-3′, 5′-ATT CGA AAG CGA AAC TAC GTA GAG GAT GAT GCA GTC TTC-3′, and 5′-GCA GTC TTC ATC GAA GCT GCA GTT GAA CTG CCC-3′ to generate K313E, R319E/R320E, K345E, R384E/R385E, K400E, K419E, R452E, and R459E mutant TRAF4-expressing constructs, respectively (QuikChange site-directed mutagenesis kit, Agilent).

To generate shT4-insensitive constructs, TRAF4 expression vectors were mutated by site-directed mutagenesis using the synthetic oligonucleotide 5′-CAA GTT CAT CTC CCA CCA GGA TAT CAG GAA AAG GAA CTA TGT GCG-3′.

### Production and Purification of Recombinant Proteins

Proteins were expressed overnight in *E. coli* BL21 (DE3) at 16°C in the presence of 0.4 mM isopropyl-β-D-thiogalactopyranoside (IPTG) (Sigma). The cell pellet was lysed and sonicated in 50 ml of Lysis buffer (50 mM NaH_2_PO_4_/Na_2_HPO_4_, 300 mM NaCl, 10 mM imidazole [pH 8.0]) containing EDTA-free complete protease inhibitor tablets (Roche). The lysate was centrifuged at 10,000× *g* for 1 h at 4°C. The supernatant was incubated with 1 ml of His-Select Nickel Affinity Gel (Sigma) overnight at 4°C under agitation. The resin was washed with wash buffer 1 (50 mM NaH_2_PO_4_/Na_2_HPO_4_, 300 mM NaCl, 25 mM imidazole [pH 8.0]) and wash buffer 2 (50 mM NaH_2_PO_4_/Na_2_HPO_4_, 300 mM NaCl, 40 mM imidazole [pH 8.0]). Bound proteins were eluted with elution buffer (20 mM NaH_2_PO_4_/Na_2_HPO_4_, 250 mM imidazole [pH 7.4]). Eluted fractions were dialyzed against Calmodulin Exchange buffer (10 mM Tris-HCl, 150 mM NaCl, 1 mM Mg(CH_3_COO)_2_, 1 mM imidazole, 10 mM β-mercaptoethanol, 0.1% NP40, 2 mM CaCl_2_, complete protease inhibitor (Roche) [pH 8]). Dialyzed fractions were incubated with 1 ml of Calmodulin Affinity Resin (Stratagene), supplemented with 3 µl of 2 M CaCl_2_ and then incubated overnight at 4°C under agitation. The resin was washed three times with Calmodulin Exchange buffer. Bound proteins were eluted with Calmodulin Elution buffer (10 mM Tris-HCl, 150 mM NaCl, 1 mM Mg(CH_3_COO)_2_, 1 mM imidazole, 10 mM β-mercaptoethanol, 0.1% NP40, 2 mM EGTA [pH 8]). The fractions were then analyzed by SDS-PAGE followed by Coomassie Blue or Western blotting.

### Coomassie Blue Staining and Immunoblotting

Proteins were assayed by BCA protein assay (Pierce). Equal amounts of protein lysates in Laemmli were loaded onto 10% SDS-PAGE gel electrophoresis. For Coomassie Blue staining, the gel was incubated in Coomassie brilliant blue R 250 (Merck) solution. For immunoblotting, the SDS-PAGE gel was transferred onto nitrocellulose membranes. Membranes were blocked for 1 h at room temperature with PBST– 4% non-fat dry milk and then incubated overnight with the following antibodies: rabbit polyclonal anti-STARD3 (IGBMC [80]), mouse anti–β-actin (A5441; Sigma), mouse anti–β-catenin (610153, BD Transduction Laboratories), rat anti-E-Cadherin (ECCD-2; 13-1900; Invitrogen), mouse anti-Desmoplakin 1/2 (2.15; Progen Biotechnik), and mouse monoclonal anti-TRAF4 (2H1; Euromedex/IGBMC). The anti-STARD3 antibody does not recognize recombinant proteins, but the immunoglobulin-binding domain of the protein A within the TAP-tag directly binds the antibody. Secondary antibodies coupled with HRP (Jackson ImmunoResearch Laboratories, Inc.) were incubated for 1 h, and antibody binding was detected by ECL (Thermo Fisher Scientific).

### Lipid Overlay Assay

Binding of recombinant proteins flanked by the TAP and the 6His tags to PIP (Phosphatidylinositol Phosphate) strips (Echelon Biosciences) or to homemade lipid-coated membranes was done as described by Dowler and collaborators [81]. Briefly, lyophilized lipids (phosphatidylethanolamine (Avanti: 850757P), PA (Avanti: 840875P), PI(3,5)P2 (Avanti: 850154P), PI(4,5)P2 (Avanti: 850155P), and PI(3,4,5)P3 (Avanti: 850156P)) were reconstituted to 1 mM stocks in a 1∶1 solution of methanol and chloroform. Lipids were diluted in a 2∶1∶0.8 solution of methanol∶chloroform∶water to 500 µM. We spotted 1 µl aliquots of each lipid and solvent onto hybond-C extra membrane (Amersham). The membranes were then dried at room temperature for 1 h. Recombinant proteins (10 nM) were incubated for 1 h at room temperature (RT) with the preblocked PIP strips or homemade lipid-strip membranes. Membranes were then washed 10 times with TBS containing 0.1% Tween-20 (TBST) and incubated for 1 h at RT with the rabbit polyclonal anti-STARD3 antibody (1/1,000; IGBMC). After 10 washes with TBST, membranes were incubated for 1 h with a peroxidase-conjugated affinipure goat anti-rabbit IgG (1∶10,000; Jackson ImmunoResearch). After 10 washes with TBST, bound proteins were detected by ECL (Thermo Fisher Scientific).

### Recombinant TRAF-6His Purification and Mass Spectrometry Analysis

For mass spectrometry analysis, recombinant proteins were expressed as 6His fusion proteins and purified with His-Select Nickel Affinity Gel (Sigma) as described above. The protein was finally purified by gel filtration over a 16/60 Superdex 200 Column (GE Healthcare) in Ammonium bicarbonate buffer (100 mM NH_4_HCO_3_ [pH 8]). Fractions containing recombinant TRAF domain were pooled and concentrated with Amicon Ultra-15 Centrifugal Filter Unit (Merck Millipore). Recombinant TRAF-6His protein was incubated in the presence or absence of a soluble form of PI(3,4,5)P3 (PI(3,4,5)P3-diC4; Echelon Biosciences) in a 1∶5 protein∶lipid ratio and then submitted to ESI-TOF (MicrO-Tof II, Bruker, Bremen, Germany). The analysis of TRAF alone and TRAF incubated with PI(3.4.5)P3 was performed in native condition with a final concentration of 20 pmol/µL of protein in ammonium bicarbonate buffer. The samples were continuously infused into the ion source at a flow rate of 3 µL/min using a syringe pump (KD Scientific, Holliston, MA). The data were acquired in the positive mode. The calibration of the device was performed with a solution of cesium iodide (Fluka) 1 mg/mL in ethanol. To preserve the noncovalent complexes, relatively mild interface conditions were used, a declustering voltage (Capillary exit) was fixed at 200 V, and the capillary temperature was set to 160 and 180°C. The time of acquisition was between 1.5 min and 2 min. Data were analyzed with Data Analysis software (Bruker) and the multicharged spectra obtained were then deconvoluted with Maximum Entropy software.

### Liposome Flotation Assay

Liposome flotation assays were performed as described in Manneville et al. [43]. Liposomes were made with DOPC (Avanti Polar Lipids, 850375C), 1% NBD-PE (Invitrogen), with or without 5% phosphinositides: 18∶1 PI(3,4,5)P3 (Avanti Polar Lipids, 850156P) or 18∶1 PI(4,5)P2 P3 (Avanti Polar Lipids, 850155P). Lipids in chloroform were mixed and the solvent was removed by evaporation. The lipid film was resuspended in HK buffer (50 mM Hepes pH 7.2, 120 mM potassium acetate). Liposomes were extruded with a mini-extruder equipped with a 100-nm pore filter (Avanti Polar Lipids). Recombinant proteins were incubated 10 min with liposomes in HKM buffer (HK supplemented with 1 mM MgCl_2_) in a total volume of 150 µl. The mix was adjusted to 30% sucrose by adding 100 µL of 2.2 M sucrose in HKM buffer. This sucrose cushion was overlayed with 200 µl HK containing 0.75 M sucrose and then 50 µl HK. The samples were centrifuged at 240,000× *g* for 1 h in a swing rotor. The bottom (200 µl), middle (200 µl), and top (100 µl) fractions were manually collected from the bottom. Liposome flotation was verified by detecting NBD-PE fluorescence: dot blots of each fraction were analyzed using a Fuji LAS-4000 fluorescence imaging system. Proteins were analyzed by SDS-PAGE followed by Western blot using an anti-His antibody (HIS-1G4, Euromedex/IGBMC). The amount of membrane-bound proteins was determined by comparing proteins present in the top fraction to a reference lane containing the total amount of the loaded protein.

### Circular Dichroism

All the CD experiments were recorded by using a Jasco J-815 spectropolarimeter (Easton, MD) fitted with an automatic six-position Peltier thermostated cell holder. The instrument was calibrated with 10-camphorsulphonic acid. Far-UV CD data were obtained using a 0.1 mm pathlength cell (Quartz-Suprasil, Hellma UK Ltd) at 25.0°C±0.1°C. Spectra were acquired using a continuous scan rate of 50 nm/min and are presented as an average of at least 20 successive scans. The response time and the bandwidth were 1 s and 1 nm, respectively. The absorbance of the sample (at a concentration of 35 µM) and buffer (Cl^−^-free buffer) was kept as low as possible. Spectra were carried out in 100 mM ammonium bicarbonate (pH 7.0) and recorded between 180 and 260 nm. All spectra were corrected by subtracting the corresponding solvent spectrum obtained under identical conditions. The signal is expressed in mean residue ellipticity (deg cm^2^ dmol^−1^). Data were deposited on the Protein Circular Dichroism Data Bank (http://pcddb.cryst.bbk.ac.uk) [82] under accession numbers CD0004232000 (TRAF-6HIS), CD0004233000 (TRAF-6HIS K413E), and CD0004234000 (TRAF-6HIS K345E).

### Dynamic Light Scattering

DLS experiments were carried with the Dynapro Nanostar instrument (Wyatt Technology). Laser wavelength was 658 nm. DLS measurements were performed at 25°C using 20 µM protein in 10 mM Tris pH 7.5, 150 mM NaCl. Each measurement was an average of 10 runs, 7 s each. Size distribution by percentage of mass (% Mass) was used for the results analysis. Datasets obtained were analyzed using the Dynamics software (Wyatt Technology).

### ITC

ITC was carried out using an ITC 200 calorimeter (Microcal, Northhampton, MA) at 25°C. The recombinant TRAF-6His protein was dialyzed extensively against 10 mM Tris pH 7.5, 150 mM NaCl. A typical titration consisted of injecting incrementally 2 µl of 500 µM inositol-(1,3,4,5)-tetrakisphosphate (IP4, Echelon) into the 16 µM protein sample, at time intervals of 3 min, to ensure that the titration peak returned to the baseline. Calorimetric data were analyzed with the evaluation software MicroCal ORIGIN (MicroCal Software, Northhampton, MA).

### Crystallization, Data Collection, and Structure Resolution

TRAF4 was crystallized at 6.4 mg ml^−1^ with a 1∶1 molar ration of inositol-(1,3,4,5)-tetrakisphosphate (IP4). The crystallization experiments were carried out by the sitting-drop vapour diffusion method at 293 K using a Cartesian nanolitre dispensing robot. A mixture consisting of 0.2 µl protein solution and 0.2 µl reservoir solution was equilibrated against 50 µl of reservoir solution. Several commercially available screens were used including the PEGs suite and the ProComplex suite (Qiagen) and Wizard I & II (Emerald Biosystems). Crystals appeared in several conditions with the best being 15% PEG 4000, 0.1 M HEPES pH 7.0. The crystals were briefly transferred to crystallization solution supplemented with 25% PEG 400 and flash cooled in liquid nitrogen.

Data were collected from a single cryo-cooled crystal (100 K) on a MarMOSAIC 225 CCD detector (Marresearch) on the ID23-2 beamline of the European Synchrotron Radiation Facility (ESRF). We collected 180° of data to 1.85 Å using 2.25° rotation and 1.55 s exposure time per image. The data were indexed and processed with XDS [83] and scaled by AIMLESS [84],[85] from the CCP4 suite [86]. The crystals belonged to the space-group P2_1_ with unit cell parameters a = 54.625 Å, b = 85.443 Å, c = 61.646 Å, β = 108.076°. The structure was solved by molecular replacement using PHASER [87] in the PHENIX suite [88]. The structure of the trimer of human TRAF2 was modified using CHAINSAW [89] to trim the side chains to the last common atom and was used as a search model. The asymmetric unit contains one copy of the TRAF4 homotrimer with a corresponding Matthews coefficient [90] of 2.02 Å^3^/Da and a solvent content of 39.2% (assuming a partial specific volume of 0.74 ml g^−1^). Refinement was performed using BUSTER (BUSTER-TNT 2.10) followed by iterative model building in COOT [91].

The quality of the refined model was assessed using MOLPROBITY [92] and Procheck [93]. Data collection and refinement statistics are summarized in Table S1. Molecular graphic figures were generated using PyMOL [94]. Coordinates and structure factors have been deposited at the Protein Data Bank with accession code 3ZJB.

### Docking of PI(3,4,5)P3-diC4 to Human TRAF4

The 3D structure of PI(3,4,5)P3 was obtained from a 2D sketch by Corina [95], and its protonation state at pH 7.4 was predicted using the Filter program [96]. A fully deprotonated form (net charge of −7) was predicted to be the most abundant species and further considered for docking. Hydrogen atoms were added to the X-ray structure of human TRAF4 by means of the SYBYL X-2.0 package [97].

The ligand was then docked into the X-ray structure of human TRAF4 with the program GOLD [98]. The active site was defined by any residue within a 10-Å-radius sphere centered at the mid-distance between Lys313A and Lys345C Cα atoms. Five residue side chains (Lys313A, Arg297C, Glu298C, Glu300C, and Lys345C) were considered flexible during the docking using the GOLD default rotamer library. Hydrogen bonding of the ligand to Lys313A and Lys345 NZ atoms was set as a prerequisite using a constraint weight of 20.0 and a minimum geometry weight of 0.005. The best docking pose of each of 20 independent docking runs was saved, therefore leading to 20 possible docking solutions, out of which the one with the best docking score was retained. The ligand topology was parameterized with the Antechamber [99] module of AMBER [100]. The corresponding protein—ligand complex was embedded in a box of 23668 TIP3P water molecules and was further refined in AMBER [100] using the AMBER general atom force field (GAFF) for the ligand and the ff99 force-field for the protein. The fully hydrated complex was refined by 1,500 steps of steepest descent plus 4,000 steps of conjugate gradient energy minimization.

### Immunofluorescence

MCF7, COS-7, and MCF10A cells were grown on glass coverslips and transfected for 24 h. Cells were then fixed for 20 min at RT with 4% paraformaldehyde in phosphate buffered saline (PBS) and permeabilized for 10 min with 0.1% Triton X-100 in PBS. After blocking with 1% bovine serum albumin (BSA) in PBS, cells were incubated at RT with the primary antibodies rabbit anti-Flag (1∶2,000; F-7425 Sigma) and mouse anti-ZO-1 (1∶1,000; Zymed). After three washes in PBS, cells were incubated for 1 h with Cy3- and Alexa488-conjugated secondary antibodies (Jackson ImmunoResearch and Invitrogen-Molecular Probes, respectively). After two washes, nuclei were counterstained with Hoechst-33258 dye. Slides were mounted in ProLong Gold (Invitrogen). Observations were made with a confocal microscope (Leica SP2 UV, 63×, NA 1.4). For TJ recruitment analysis, images were acquired using the same confocal microscope settings (laser intensity, PMT gain …). The overlapping staining between WT or mutant TRAF4 and ZO-1 was highlighted in white by the “colocalization analysis” tool from ImageJ software (http://rsbweb.nih.gov/ij/). Pixels were considered colocalized if their intensity value was above the threshold value (50) and the ratio of their intensity higher than 50%. A colocalization index corresponding to the overlapping area between TRAF4 and ZO-1 divided by the TJ length was then calculated. Ten fields per condition were acquired for the quantification. For the measurement of TJ formation and/or stability, immunofluorescence was performed 48 h after MCF10A cell line plating as described above.

### Calcium-Switch Assay

Calcium switch experiments were performed with minor modifications of previously published methods [101],[102]. In brief, MCF7 cells were plated at high density in DMEM supplemented with 10% FCS, 0.6 µg/ml insulin, and 40 µg/ml gentamycin (HCM). After they reached confluence, cells were washed twice with the EMEM low-calcium medium (EMEM-LCM) and incubated with EMEM-LCM for 16 h. Cells were then switched back to HCM at *t* = 0, and junctional reassembly was followed for various times.

### Boyden Chamber Assay

The lower Transwell chamber (Millicell, Merck Millipore) contained cell-appropriate medium (10% FCS, 2% BSA as chemoattractants). Cells in appropriate medium (0.2% BSA) were seeded onto membranes of the upper Transwell chamber (6.5 mm diameter, 8 µm pores), MCF10A (40×10^4^ cells, 16 h) and MCF7 (10^5^ cells, 48 h). After incubation, cells were ethanol-fixed and nuclei were counterstained with Hoechst-33258 dye. Cells at the membrane upper face were scraped, and those of the lower face were counted after acquisition using an inverted microscope. A total of 36 microscopic fields from three independent experiments were used for quantification. Nuclei were counted with the “nucleus counter” ImageJ plugin.

### Statistical Analysis

Averages and standard deviations are shown in the graphs in Figures 1E, 7C,F, 8C, S2C, and S6C. Analyses were performed by a one-way ANOVA test, followed by the Dunnet's multiple comparison test (GraphPad Prism). *p<0.05, **p<0.01, and ***p<0.001.

## Supporting Information

Figure S1 **TRAF4 does not impact on adherens junctions in MCF10A cells.** (A) Western blot analysis of adherens junction (AJ), desmosome, and TJ proteins in parental and in established MCF10A cell lines (Figure 1A). (B–D) The presence of AJ was estimated by the presence of membrane-bound β-catenin staining in the different cell lines of TRAF4 loss of function (B), gain of function (C), and rescue experiments (D). Left panels are representative confocal sections of β-catenin staining (green), and right panels are merges with Hoechst staining (blue). Scale bar, 20 µm.
(TIF)

Figure S2 **TRAF4 knock-down accelerates TJ reassembly in MCF7 cells.** (A) Western blot analysis of TRAF4 expression. In MCF7 cells, TRAF4 expression has been silenced (lanes 3–5) and restored in silenced cells (lane 5). Parental (lane 1) and control sh (lane 2) together with a TRAF4-silenced line transduced with the empty vector (lane 4) were used as controls. TRAF4 expression levels were normalized to control parental cells using β-actin as loading control; values are indicated on the top. (B) Calcium switch assay in MCF7 cells. This assay involves the disruption of epithelial junctions by extracellular calcium removal followed by a rapid reassembly triggered by calcium repletion. Representative confocal image of ZO-1 staining at 0, 3, 5, 7, and 20 h after calcium repletion are shown from left to right, respectively (inverted grey look-up table). (C) TJ quantification at 3, 5, and 7 h after calcium repletion. Score representing the number of cells with a continuous ZO-1 staining, normalized to parental MCF7 cells (percentage). Ten microscopic fields were used for the quantification.
(TIF)

Figure S3 **PIP binding of the TRAF domain is conserved through evolution.** (A) Coomassie blue staining (a) and Western blot analysis (b) of purified recombinant TRAF domains of human and fly TRAF4 (dTRAF1). (B) Lipid-overlay assay of TRAF domains from human and fly TRAF4. In this assay, the TAP-6His and the TRAF domain of human TRAF4 are used as the negative and positive control, respectively. Immunodetection of membrane-bound proteins was performed as described in Figure 2C. Please note that dTRAF1 binds to PIPs similarly to the human TRAF4.
(TIF)

Figure S4 **Crystal structure representing exposed basic residues of the TRAF domain of TRAF4 selected for mutagenesis.** Top view (left) and bottom view (right) of the surface representation of the TRAF domain of TRAF4. The three TRAF monomers are colored in magenta, cyan, and green, respectively. Surface-exposed basic residues selected for the mutagenesis assay are colored in red.
(TIF)

Figure S5 **The TRAF-K345E mutant is trimeric.** The quaternary structures of wild-type and K345E TRAF4-TRAF domains were analyzed by gel filtration (A) and dynamic light scattering (B) experiments. (A) Gel filtration was performed with 1 ml containing 1 mg and 0.3 mg of WT and K345E mutant TRAF domains, respectively. Both WT and mutant TRAF domains eluted in the same fractions, which indicates that their sizes are similar. (B) Dynamic light scattering performed using 20 µM WT and K345E mutant TRAF domain of TRAF4 indicated that both WT and mutant TRAF domains have similar radii.
(TIF)

Figure S6 **TRAF4 stimulates migration of MCF10A cells.** (A) Western blot analysis of TRAF4 expression. In MCF10A cells, TRAF4 expression has been silenced (lanes 2–6), increased (lane 8), and restored in silenced cells using the WT (lane 5) and the K345E mutant (lane 6). Parental (lane 1), control shRNA (lane 2), and control expression vector (lane 7) together with a TRAF4-silenced line transduced with the empty vector (lane 4) were used as controls. TRAF4 expression levels were normalized to control parental cells using β-actin as loading control; values are indicated on the top. (B) Representative microscopic field of the bottom side of the transwell. Migrating cell nuclei were stained with Hoechst, and images are shown as inverted look-up table. (C) Bar chart representing the quantification of cell migration in MCF10A cells. The number of cells that migrated were counted and normalized to control parental cells. Thirty-six microscopic fields from three independent experiments were used for the quantification.
(TIF)

Table S1 **Data collection and refinement statistics.** Values in parentheses are for the outermost resolution shell.
(DOC)

## Abbreviations

AJ: adherens junction
MEC: mammary epithelial cell
PAR3: partitioning-defective 3
PH: pleckstrin homology
PIP: phosphoinositide
TJ: tight junction
TRAF: tumor necrosis factor receptor-associated factor
ZO-1: zonula occludens 1
