# Supplementary figures and images for: TRAF4 Is a Novel Phosphoinositide-Binding Protein Modulating Tight Junctions and Favoring Cell Migration
Source: PLoS Biol. 2013 Dec 3;11(12):e1001726. doi: 10.1371/journal.pbio.1001726 (PMC3848981; doi:10.1371/journal.pbio.1001726)

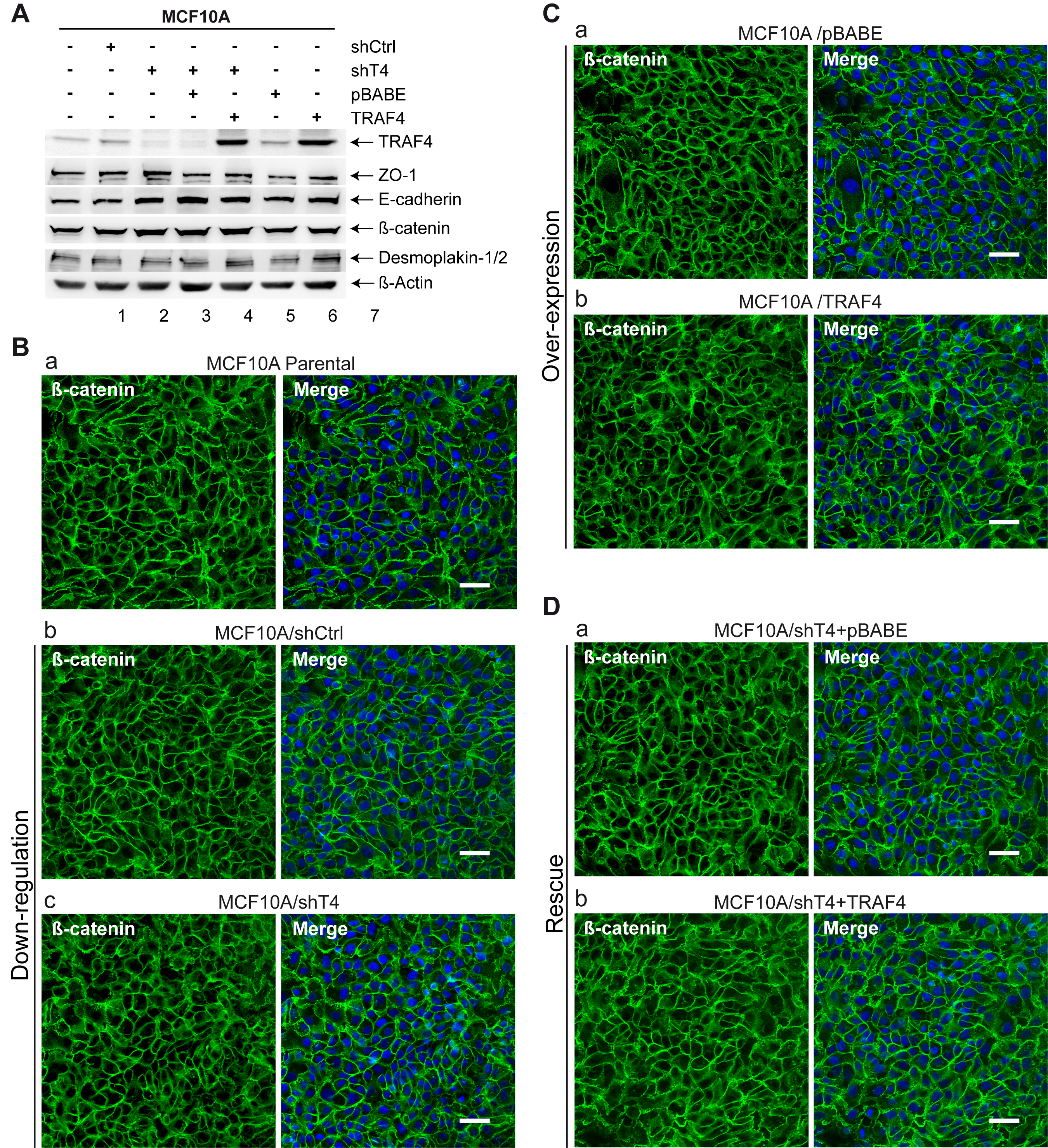

Supplement: Figure S1 — TRAF4 does not impact on adherens junctions in MCF10A cells. (A) Western blot analysis of adherens junction (AJ), desmosome, and TJ proteins in parental and in established MCF10A cell lines (Figure 1A). (B–D) The presence of AJ was estimated by the presence of membrane-bound β-catenin staining in the different cell lines of TRAF4 loss of function (B), gain of function (C), and rescue experiments (D). Left panels are representative confocal sections of β-catenin staining (green), and right panels are merges with Hoechst staining (blue). Scale bar, 20 µm. (TIF) [file pbio.1001726.s001.tif]

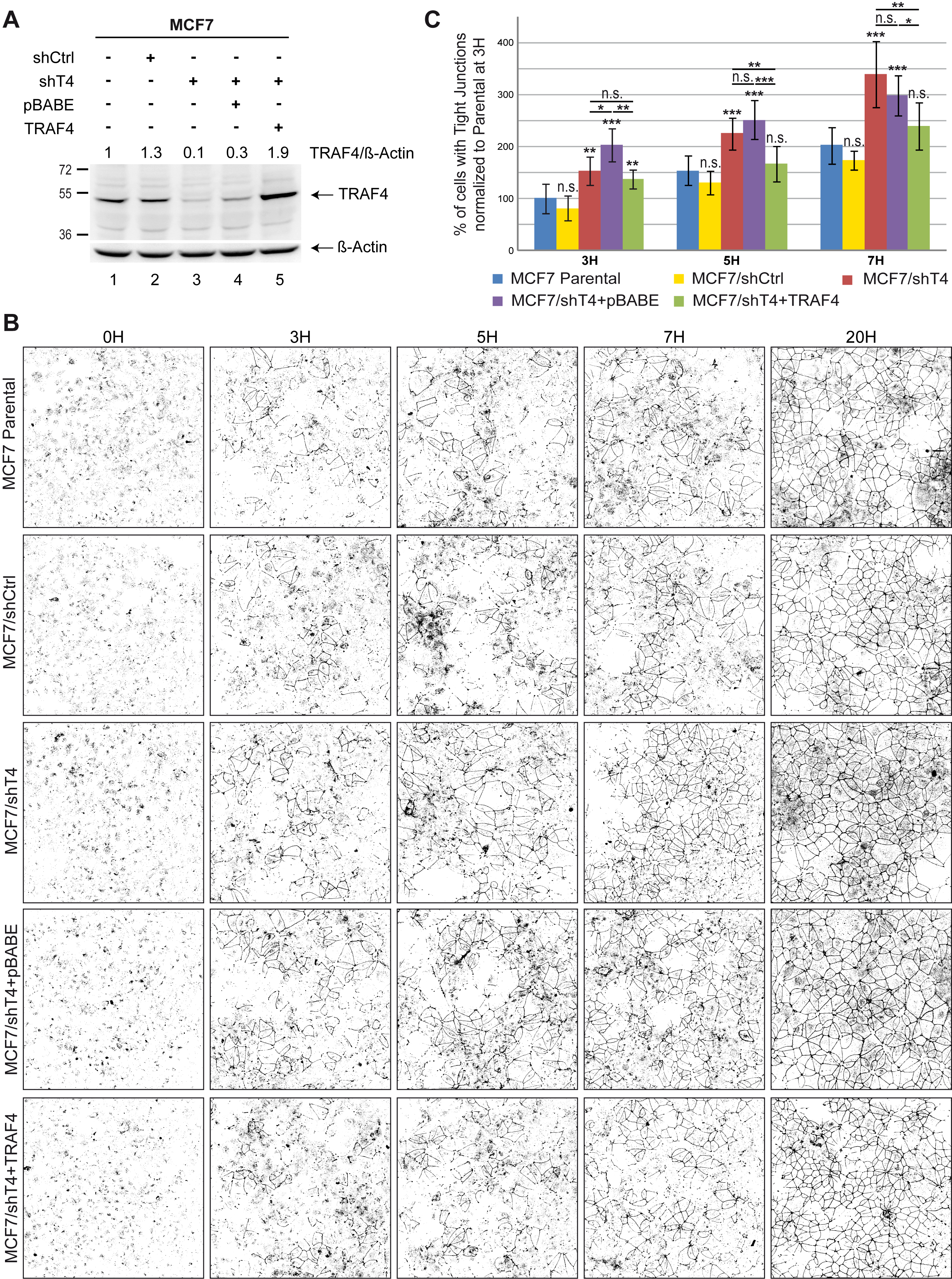

Supplement: Figure S2 — TRAF4 knock-down accelerates TJ reassembly in MCF7 cells. (A) Western blot analysis of TRAF4 expression. In MCF7 cells, TRAF4 expression has been silenced (lanes 3–5) and restored in silenced cells (lane 5). Parental (lane 1) and control sh (lane 2) together with a TRAF4-silenced line transduced with the empty vector (lane 4) were used as controls. TRAF4 expression levels were normalized to control parental cells using β-actin as loading control; values are indicated on the top. (B) Calcium switch assay in MCF7 cells. This assay involves the disruption of epithelial junctions by extracellular calcium removal followed by a rapid reassembly triggered by calcium repletion. Representative confocal image of ZO-1 staining at 0, 3, 5, 7, and 20 h after calcium repletion are shown from left to right, respectively (inverted grey look-up table). (C) TJ quantification at 3, 5, and 7 h after calcium repletion. Score representing the number of cells with a continuous ZO-1 staining, normalized to parental MCF7 cells (percentage). Ten microscopic fields were used for the quantification. (TIF) [file pbio.1001726.s002.tif]

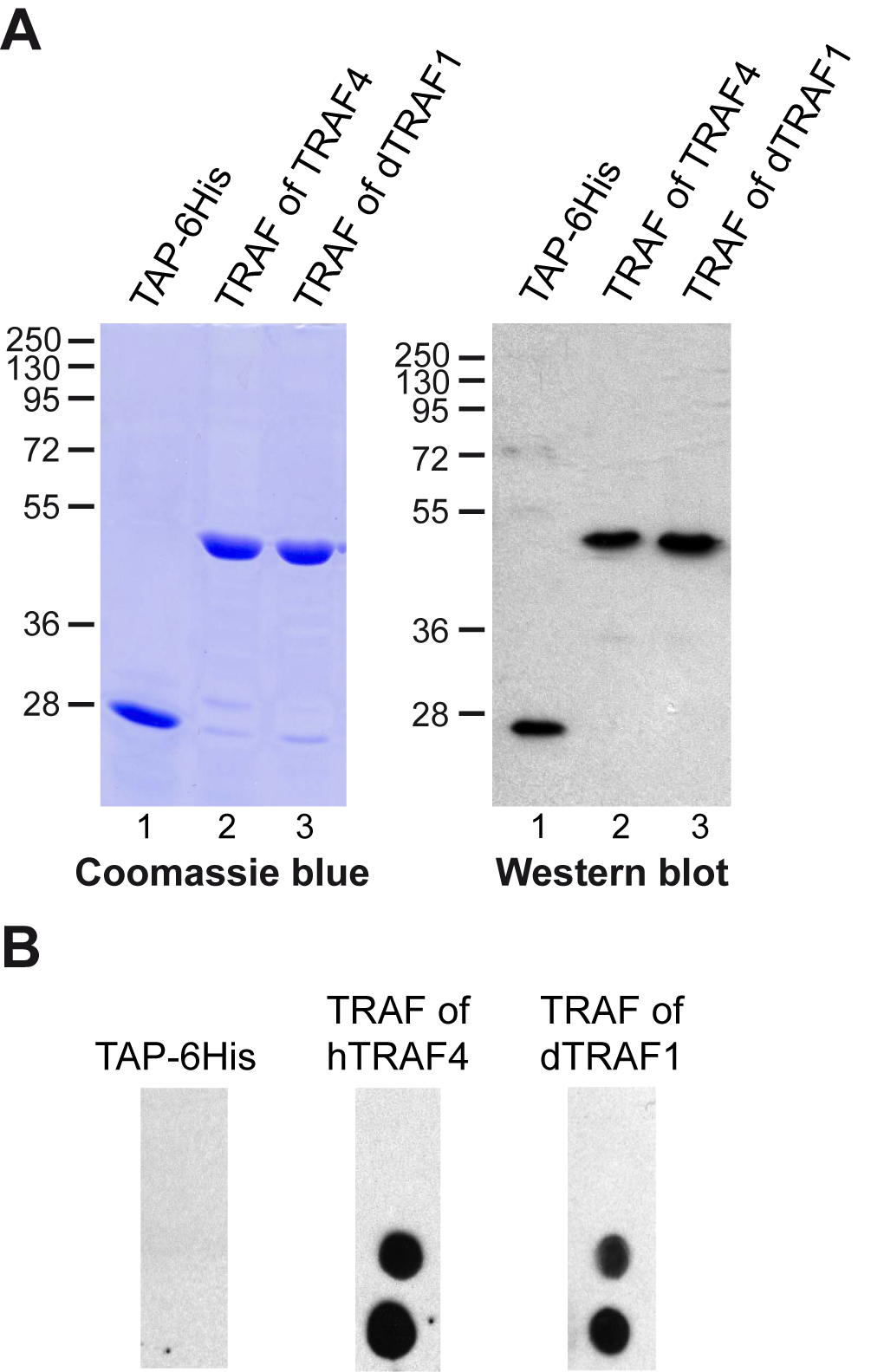

Supplement: Figure S3 — PIP binding of the TRAF domain is conserved through evolution. (A) Coomassie blue staining (a) and Western blot analysis (b) of purified recombinant TRAF domains of human and fly TRAF4 (dTRAF1). (B) Lipid-overlay assay of TRAF domains from human and fly TRAF4. In this assay, the TAP-6His and the TRAF domain of human TRAF4 are used as the negative and positive control, respectively. Immunodetection of membrane-bound proteins was performed as described in Figure 2C. Please note that dTRAF1 binds to PIPs similarly to the human TRAF4. (TIF) [file pbio.1001726.s003.tif]

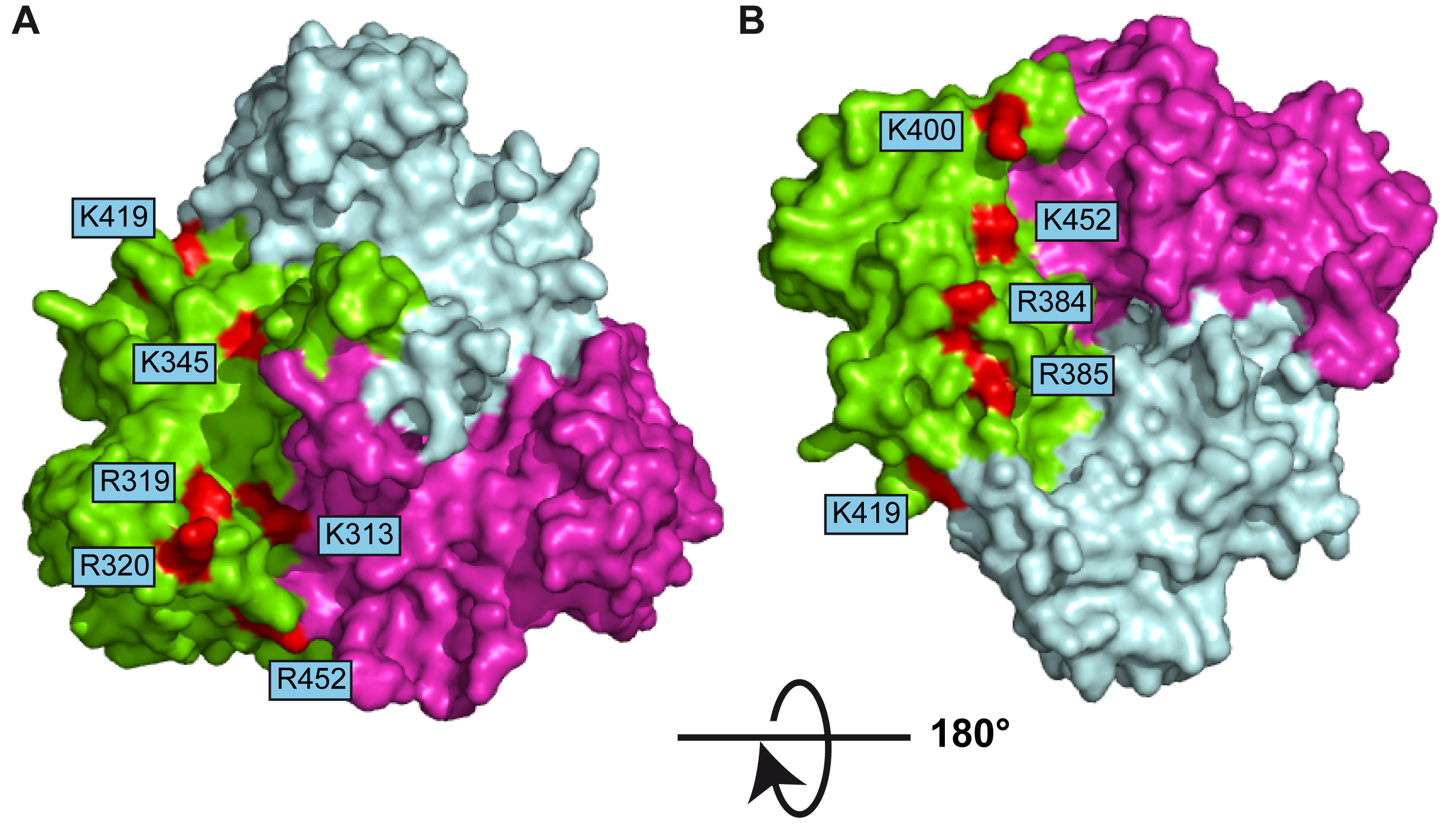

Supplement: Figure S4 — Crystal structure representing exposed basic residues of the TRAF domain of TRAF4 selected for mutagenesis. Top view (left) and bottom view (right) of the surface representation of the TRAF domain of TRAF4. The three TRAF monomers are colored in magenta, cyan, and green, respectively. Surface-exposed basic residues selected for the mutagenesis assay are colored in red. (TIF) [file pbio.1001726.s004.tif]

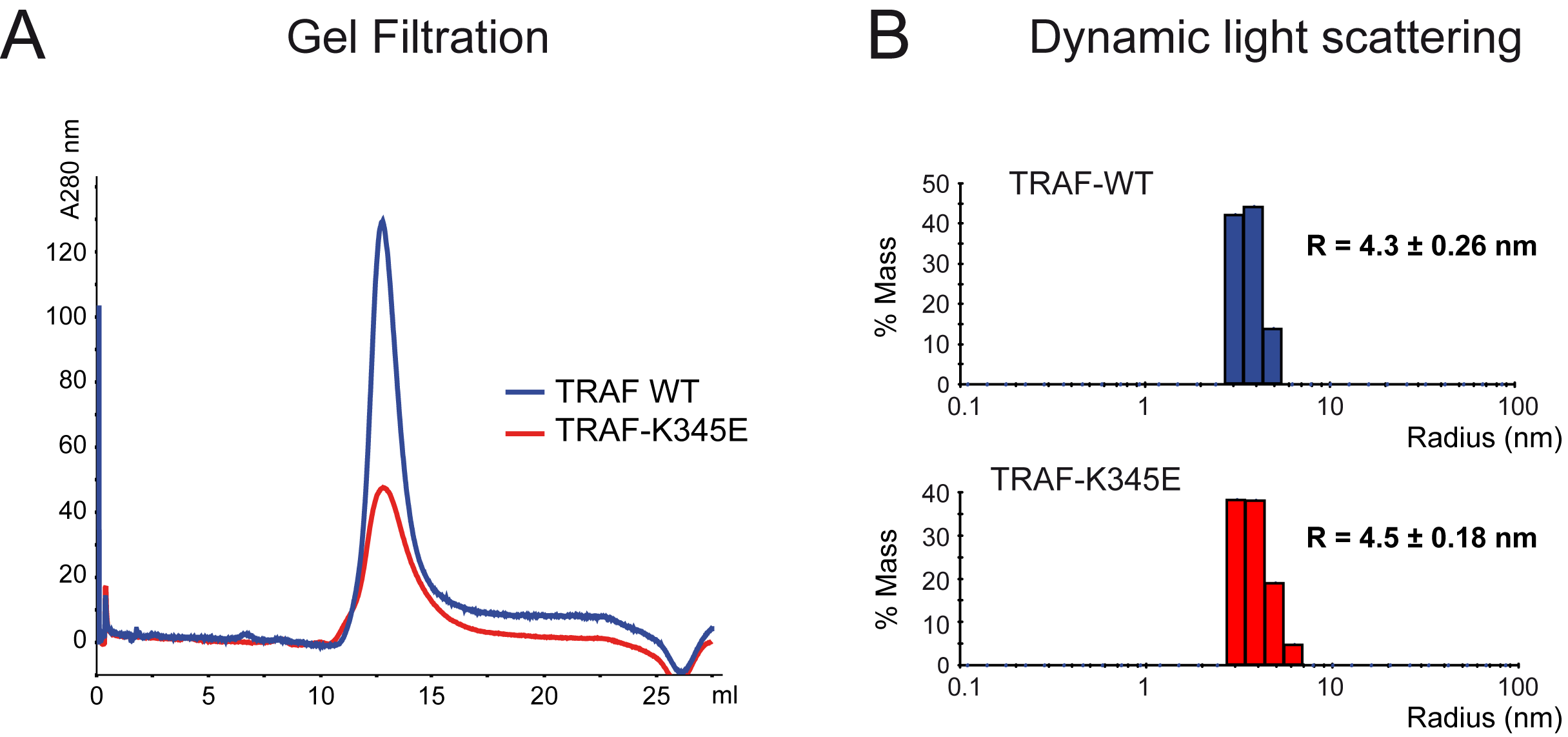

Supplement: Figure S5 — The TRAF-K345E mutant is trimeric. The quaternary structures of wild-type and K345E TRAF4-TRAF domains were analyzed by gel filtration (A) and dynamic light scattering (B) experiments. (A) Gel filtration was performed with 1 ml containing 1 mg and 0.3 mg of WT and K345E mutant TRAF domains, respectively. Both WT and mutant TRAF domains eluted in the same fractions, which indicates that their sizes are similar. (B) Dynamic light scattering performed using 20 µM WT and K345E mutant TRAF domain of TRAF4 indicated that both WT and mutant TRAF domains have similar radii. (TIF) [file pbio.1001726.s005.tif]

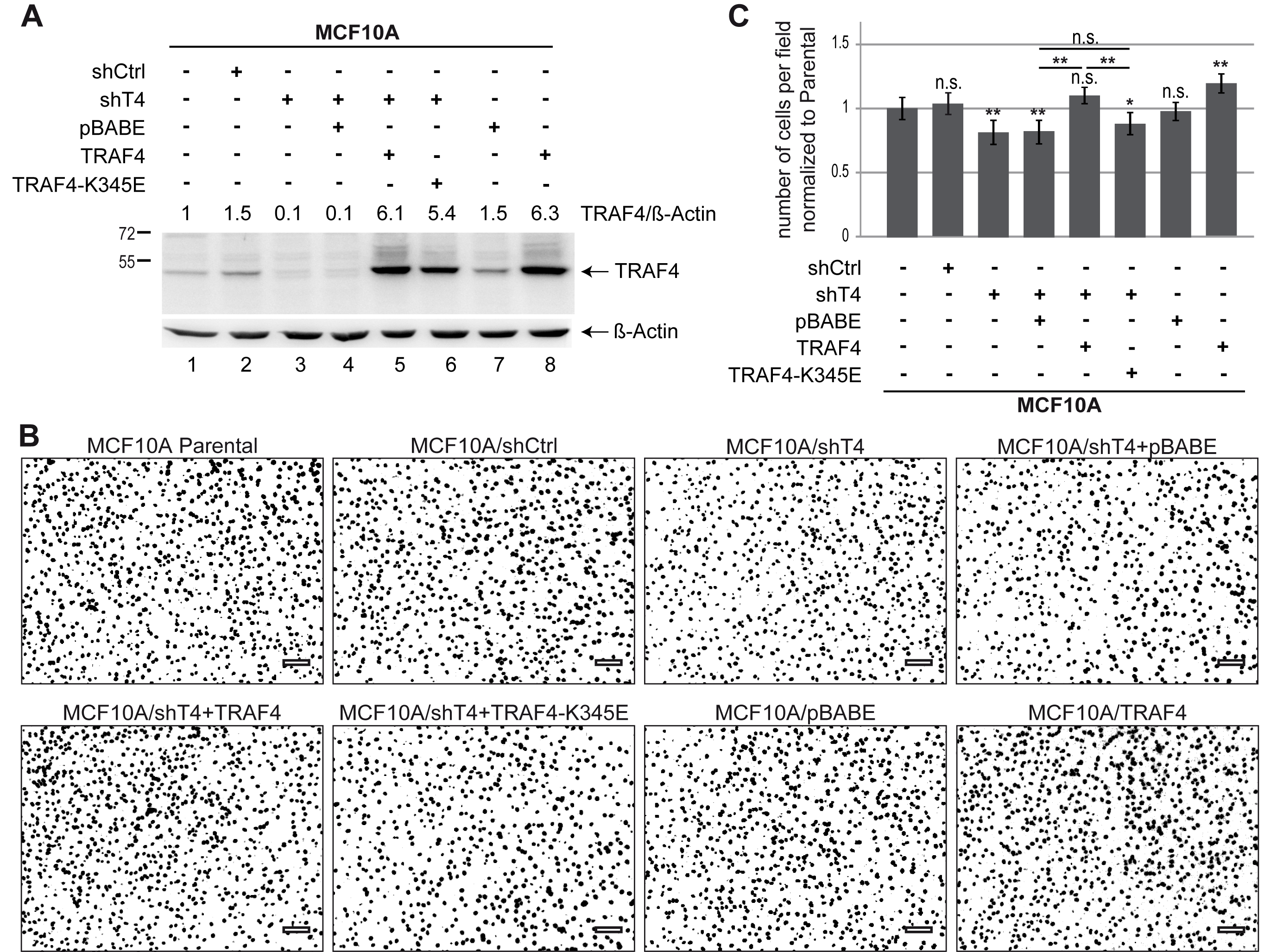

Supplement: Figure S6 — TRAF4 stimulates migration of MCF10A cells. (A) Western blot analysis of TRAF4 expression. In MCF10A cells, TRAF4 expression has been silenced (lanes 2–6), increased (lane 8), and restored in silenced cells using the WT (lane 5) and the K345E mutant (lane 6). Parental (lane 1), control shRNA (lane 2), and control expression vector (lane 7) together with a TRAF4-silenced line transduced with the empty vector (lane 4) were used as controls. TRAF4 expression levels were normalized to control parental cells using β-actin as loading control; values are indicated on the top. (B) Representative microscopic field of the bottom side of the transwell. Migrating cell nuclei were stained with Hoechst, and images are shown as inverted look-up table. (C) Bar chart representing the quantification of cell migration in MCF10A cells. The number of cells that migrated were counted and normalized to control parental cells. Thirty-six microscopic fields from three independent experiments were used for the quantification. (TIF) [file pbio.1001726.s006.tif]
