# Supplementary material for: TRAF4 Is a Novel Phosphoinositide-Binding Protein Modulating Tight Junctions and Favoring Cell Migration
Source: PLoS Biol. 2013 Dec 3;11(12):e1001726. doi: 10.1371/journal.pbio.1001726 (PMC3848981; doi:10.1371/journal.pbio.1001726)
Supplement: Table S1 — Data collection and refinement statistics. Values in parentheses are for the outermost resolution shell. (DOC) [file pbio.1001726.s007.doc]

| PDB code | 3ZJB |
| --- | --- |
| Data collection |  |
| Beamline | ID23eh2, ESRF |
| Space group | P 21 |
| Unit cell dimensions | a = 54.62Å, b = 85.44Å, c = 61.65Å, β = 108.08° |
| Resolution (Å) | 58 – 1.85 (1.88 – 1.85) |
| Rmerge † | 0.085 (1.289) |
| CC ½ (%) | 99.6 (45.7) |
| I/σ(I) | 10.8 (1.04) |
| Completeness (%) | 97.3 (73.9) |
| No. reflection | 162719 (4922) |
| No. unique reflections | 45129 (2114) |
| Multiplicity | 3.6 (2.3) |
| Wilson B | 20.8 |
| Refinement |  |
| R_work_/R_free_ | 0.1632 / 0.1995 |
| No. atoms | 4932 |
| Protein | 4354 |
| Ion | 3 |
| Water | 575 |
| B factors (Å²) |  |
| Protein | 30.57 |
| Ion | 55.24 |
| Water | 39.47 |
| R.m.s.d. from ideal geometry |  |
| Bond lengths (Å) | 0.010 |
| Bond angles (°) | 1.04 |
| Ramachandran plot (%) ‡ |  |
| Favoured | 97.3 |
| Allowed | 2.7 |
| Disallowed | 0 |
| † Rmerge = Σ*_hkl_*Σ*_i_*|*I_i_*(*hkl*) - ‹*I*(*hkl*)›|/Σ*_hkl_*Σ*_i_I_i_*(*hkl*), where ‹*I*(*hkl*)› is the average intensity of equivalent reflections. ‡ Ramachandran plot statistics were calculated by RAMPAGE (Lovell *et al*., 2003). | |

**Table S1:** Data collection and refinement statistics.

Values in parenthesis are for the outermost resolution shell.
